# Sustainable Fabrication and Transfer of High‐Precision Nanoparticle Arrays Using Recyclable Chemical Pattern Templates

**DOI:** 10.1002/advs.202407393

**Published:** 2024-12-07

**Authors:** Huaining Zha, Wenjie Zhang, Peng Chen, Jing Tao, Li Qiu, Fan Yang, Shunsheng Ye, Yutao Sang, Zhihong Nie

**Affiliations:** ^1^ State Key Laboratory of Molecular Engineering of Polymers Shanghai Key Laboratory of Metasurfaces for Light Manipulation Department of Macromolecular Science Fudan University Shanghai 200438 P. R. China; ^2^ State Key Laboratory of Surface Physics, Key Laboratory of Micro‐ and Nano‐Photonic Structures (Ministry of Education) Department of Physics Fudan University Shanghai 200433 P. R. China; ^3^ Department of Chemistry College of Sciences Northeastern University Shenyang 110819 P. R. China

**Keywords:** nanoparticle arrays, plasmonic nanoparticles, soft nanoimprinting, surface lattice resonances, template‐directed assembly

## Abstract

Nanoparticle (NP) arrays, particularly those with plasmonic properties, have diverse applications in electronics, photonics, catalysis, and biosensing, but their precise and scalable fabrication remains challenging. In this work, a facile chemical‐based strategy is presented for the fabrication of precise NP patterns using a combination of soft thermal nanoimprinting and template‐directed assembly. The approach enables the creation of well‐defined NP arrays with single‐particle resolution and yields over 99%, covering a diverse range of NP sizes from 30 to 150 nm. These patterns can be transferred onto various substrates including semiconductors, insulators, 2D materials, and flexible polymers, maintaining high uniformity and repeatability for over 60 cycles with minimal degradation. Moreover, the method enables the fabrication of extensive NP arrays up to 1 cm^2^ with a positional accuracy of ±11 nm for 30 nm NPs. As a result, the obtained silver NP arrays exhibit ultranarrow surface lattice resonances with a linewidth of 4 nm and a quality factor (*Q*) of 216. The method offers new avenues for the creation of plasmonic NP arrays for flexible and wearable devices.

## Graphical abstract

This work presents a facile chemical‐based strategy for creating centimeter‐scale, well‐defined nanoparticle arrays with single‐particle resolution. The approach enables precise fabrication, scalability, and transferability onto various substrates. The transfer process preserves chemical templates for subsequent replication‐transfer cycles. The method achieves exceptional positional accuracy and ultranarrow surface lattice resonances, offering new avenues for flexible and wearable devices.

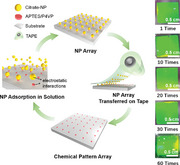

## 1 Introduction

Plasmonic nanoparticle (NP), typically composed of noble metals such as gold and silver, can efficiently concentrate and amplify incident electromagnetic fields through localized surface plasmon resonances (LSPRs).^[^ ^1^ ^]^ These nanoscale light‐matter interactions have established plasmonic NPs as a cornerstone in the field of nanophotonics.^[^ ^2^, ^3^, ^4^ ^]^ The optical properties of these NPs are largely determined by their composition, size, morphology, and surrounding dielectric environment.^[^ ^5^ ^]^ Additionally, the precise spatial organization of NPs adds another dimension for tuning plasmon modes by modulating their near‐field or far‐field interactions, resulting in unique spectroscopic phenomena.^[^ ^6^, ^7^, ^8^ ^]^ A notable examples is surface lattice resonances (SLRs), which emerge from the radiative coupling between LSPR modes and diffractive modes in long‐range ordered NP arrays.^[^ ^9^, ^10^ ^]^ This hybrid collective mode is characterized by extended resonance lifetimes, sharp spectral linewidths, and enhanced electromagnetic fields. These intriguing properties make plasmonic NP arrays highly promising for various applications such as sensors,^[^ ^11^, ^12^ ^]^ lasers,^[^ ^8^, ^13^ ^]^ nonlinear optics,^[^ ^14^ ^]^ surface‐enhanced Raman scattering (SERS),^[^ ^15^, ^16^, ^17^ ^]^ fluorescence enhancement,^[^ ^18^, ^19^, ^20^ ^]^ structural colors,^[^ ^21^, ^22^, ^23^ ^]^ and photocatalysis.^[^ ^24^ ^]^

Efficient fabricating plasmonic NP arrays at low cost and integrating them onto functional surfaces remains a significant challenge. To date, top‐down lithographic techniques have been the preferred method for preparing NP arrays due to their precise control over structures and spatial arrangement.^[^ ^25^, ^26^ ^]^ However, these approaches are criticized for their high cost, low throughput, material wastage, and stringent requirements on sophisticated facilities. Additionally, the resulting metallic arrays are typically limited to a narrow range of materials and suffer from surface roughness and grain boundaries that contribute to significant radiative losses. In contrast, colloidal NPs synthesized via wet chemical methods exhibit superior properties compared to their lithographically fabricated counterparts.^[^ ^27^, ^28^ ^]^ Efforts have been made to assemble these colloidal building blocks into desired arrays using bottom‐up approaches such as interfacial self‐assembly and template‐directed assembly.^[^ ^29^, ^30^, ^31^ ^]^ While large‐area arrays of colloidal NPs can be readily achieved through interfacial self‐assembly, limitations such as restricted lattice types (predominantly hexagonal) and defects like dislocations and vacancies hinder their practical device implementation.^[^ ^32^, ^33^, ^34^ ^]^ Moreover, colloidal building blocks are usually coated with dielectric shells (e.g., polymers) that determine the interparticle distance.^[^ ^35^ ^]^ Thus, integrating NP arrays into functional devices necessitates additional etching processes to remove the shells, which may damage or contaminate the core NPs or the target substrates. Template‐directed assembly approaches offer a promising solution by combining the advantages of top‐down and bottom‐up techniques.^[^ ^36^, ^37^, ^38^ ^]^ These methods use lithographically patterned topological or chemical templates to guide the precise placement of NPs through mechanisms such as capillary force^[^ ^39^, ^40^, ^41^, ^42^ ^]^ DNA recognition,^[^ ^43^ ^]^ electrostatic attraction,^[^ ^44^ ^]^ electrophoresis/dielectrophoresis,^[^ ^45^, ^46^ ^]^ direct growth.^[^ ^47^, ^48^ ^]^ Ma et al. developed an electrostatic funnel guiding method that offers high precision in NP placement and compatibility with CMOS technology.^[^ ^49^ ^]^ Jonas and Krüger employed photolithographic patterning on silane layers and investigated colloidal assembly behavior in both organic and aqueous media.^[^ ^50^ ^]^ Kinnear et al. utilized EBL to etch wells into PMMA films and selectively functionalized hydroxyl groups on the silicon dioxide surface, enabling efficient placement of single NPs.^[^ ^51^ ^]^ While template‐directed assembly methods offer a straightforward and efficient approach, several challenges remain. First, templates are typically designed for single‐use with specific target substrates, and they must be removed before integrating NP arrays into functional devices. This limitation hinders repeatability across multiple fabrications, which is crucial for achieving optimal performance and signal stability of NP patterns. Second, achieving a high quality factor (*Q*) value of NP patterns, which is crucial for the sensitivity and efficiency of optical devices by ensuring low energy loss and sharp resonance, is challenging. This difficulty arises mainly from the precise placement required for individual NP arrays. Third, methods such as EBL for making templates are costly, complicated, and time‐consuming, making the rapid assembly of single NPs over large areas difficult. Fourth, the size and type of the NP are difficult to change due to the inherent limitations of the template.

Here, we present a robust strategy designed for the repeatable replication of centimeter‐scale plasmonic NP arrays with high fidelity utilizing reusable templates. This strategy leverages soft thermal nanoimprinting to create chemical pattern arrays on a substrate, guiding the site‐selective adsorption of chemically synthesized colloidal NPs via electrostatic interaction. It enables the assembly of NPs, ranging from 30 to 150 nm in diameter, into scalable arrays with single‐particle resolution and yields exceeding 99%. Various ordering parameters (e.g., square, hexagonal) of NP arrays can be realized by selecting original hard master with predetermined lattice parameters. Owing to the highly ordered organization and minimal defects, the resulting NP arrays exhibit excellent optical properties. For instance, our Ag NP arrays display ultranarrow SLRs with a linewidth as narrow as ≈4 nm and a *Q* of 216. Furthermore, we utilize planar chemical pattern templates without topographical cavities, ensuring fast and intact transfer of the NP arrays using easily accessible media, such as adhesive tapes, onto various surfaces (e.g., Si, quartz, 2D materials, metals, flexible polymer films). This transfer process preserves the chemical templates for the next replication‐transfer cycle of NP arrays. We demonstrate that at least 60 replicas of NP arrays can be produced using a single template without compromising quality. Our method offers a cost‐effective and versatile solution for the fabrication of NP arrays on various surfaces, with potential applications in electronics, photonics, catalysis, structural color, and biosensing.

## 2 Results and Discussion

### 2.1 Sustainable Fabrication and Transfer of Large‐Area NP Arrays

**Figure 1:**
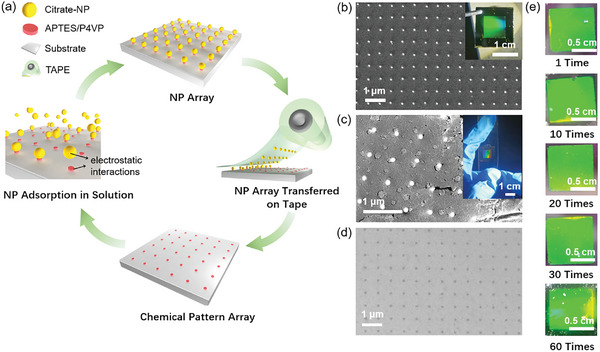
Recyclable fabrication and transfer of AuNPs. a) Schematic illustration of an assembly‐transfer cycle involving the fabrication of chemical pattern array, electrostatic interaction‐driven adsorption of NPs, and transfer of the assembled NP arrays onto a substrate. b–d) SEM images of the AuNPs pattern on a SiO_2_/Si substrate (b), on tape after the transferr process. c), and d) the P4VP array. The insets show corresponding photographs of the AuNPs pattern taken in flashlighting light. e) Photographs of the AuNPs array transferred onto a PMMA flexible film at different cycles.

The fabrication process starts with the formation of a chemical pattern array of (3‐**a**minopropyl)triethoxysilane (APTES) or poly(4‐vinylpyridine) (P4VP) self‐assembled monolayers (SAMs) on a substrate using soft thermal nanoimprinting (Figure S1, Supporting Information; see Experimental Section for details). The protonated amino group in APTES attracts negatively charged NPs, while the Si‐O‐Si covalent bond ensures robust stability for recycling the APTES pattern on oxide substrates like silica and indium tin oxide (ITO). P4VP is specifically chosen to bind noble metal substrates (e.g., Au) and metal NPs together through the interaction between transition metals and pyridine. Gold NPs (AuNPs) were selected as the model NPs due to their controllable gemometries and optical characteristics. Spherical citrate‐capped AuNPs with a mean diameter (*D*_NP_) of 100 ± 4 nm were synthesized via a seed‐mediated growth method, and dispersed in water (Figure S2, Supporting Information). In acidic solutions, the amino groups of APTES or the pendant pyridine rings of P4VP can be protonated. Thus, the electrostatic attraction starts to work between the AuNPs stabilized by carboxylic groups and the chemical patterns on the substrate, leading to efficient and selective absorption of the AuNPs onto the predefined patterns with high yield (**Figure** **1**b). The adsorption process is fast, as reflected by the appearance of structural color within 5 s (Figure S3, Supporting Information).

The moderate strength of the bonds between NPs and APTES (or P4VP) nanopatterns preserves NPs’ positions during washing and drying processes, and favors stripping them off the substrates. We chose commercial Scotch 600 transparent tape to strip an entire NP array from the original substrate. Notably, the overall AuNPs pattern retained a remarkable level of uniformity on the tape, as confirmed by the distinct structural color and scanning electron microscopy (SEM) images (Figure 1c). When the AuNPs array, delicately affixed face down to the quartz substrate using tape, the AuNPs array exhibits a sharp SLR peak ≈875 nm in the transmission spectrum. It underscored the remarkable regularity of the array—even within this challenging refractive environment (Figure S4, Supporting Information). Additionally, we explored alternative transfer media such as poly(methyl methacrylate) (PMMA). The PMMA film with a thickness of 2 µm was prepared on an AuNPs array through spin‐coating (Figure S5, Supporting Information). After immersion in a positive photoresist developer for 10 min, the AuNPs array/PMMA composite film was peeled off using tweezers, resulting in a freestanding film that can be transferred to another substrate with the assistance of water (Figure S6, Supporting Information).

It is worth noting that the complete transfer of the NP array simultaneously restored the chemical pattern template to its pristine condition, allowing for subsequent generation of a new NP array (Figure 1d). The P4VP pattern array maintained its effective adsorption capacity with a high yield of ≈95% even after 60 adsorption‐transfer cycles (Figure 1e; Figure S7, Supporting Information). The corresponding transmission spectrum of the new NP array is shown in Figure S8 (Supporting Information). Due to the approximate refractive index match between the PMMA film and the transferred quartz substrate, the SLR of (±1,±1) band‐edge mode is observable in the spectrum. The SLR peak of the 60th array exhibited an 25% decrease in intensity compared to the initial array (Figure S9, Supporting Information). The durability and repeatability of this technique were confirmed by the high‐resolution X‐ray photoelectron spectroscopy (XPS) spectra of the chemical pattern (Figure S10 and Table S1, Supporting Information). These spectra were acquired after 30 cycles with tape and showed minimal changes compared to those obtained from the initial substrate. This result suggests that the chemical template can be reused for multiple times without significant degradation. A mechanical model on the process of transferring AuNPs on P4VP with tape (Figure S11, Supporting Information) indicates that the direct adsorption of AuNPs onto the fully P4VP‐modified substrate indirectly reflects changes in P4VP density (Figure S12, Supporting Information). Specifically, after the 90th transfer, the P4VP density can be approximated to have decreased to 90% of its initial value.

### 2.2 High Tunability and Yield in the Fabrication of NP Arrays

**Figure 2:**
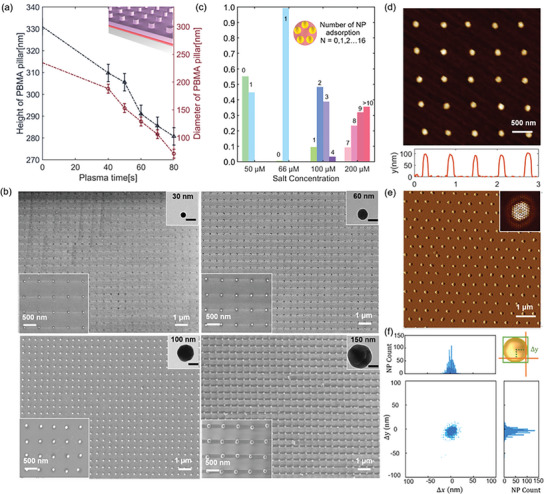
Characterizations of the AuNPs arrays. a) The height (*H*_p_, depicted in black) and diameter (*D*_p_, depicted in red) of PBMA nanocylinders vary as a function of oxygen plasma etching time. b) SEM images of NP arrays with different diameters. The insets show corresponding transmission electron microscopy (TEM) of NP. Scale bar: 30 nm. c) Effect of salt concentrations on the adsorption number (N) of 60 nm NPs on one absorption site. The inset provides an example where five NPs are simultaneously attached to one absorption site. d) AFM images and height profiles of 100 nm AuNPs arrays. e) AFM images and the corresponding FFT patterns of hexagonal AuNPs arrays. f) Distribution of individual 30 nm NPs lateral offset relative to their designated position as defined by the template. The data, collected from 1000 NPs, indicates a mean placement accuracy of ±11 nm post‐chemisorption (80% numbers).

Large‐area arrays of different‐sized NPs can be prepared by simply adjusting the duration of plasma etching and the ionic strength of NP solutions. A silicon master with nanopillars (diameter *D_0_* = 250 nm, height *H_0_* = 350 nm, period *P* = 600 nm) was utilized in a nanoimprint process, molding it against elastomeric polydimethylsiloxane (PDMS) to create soft PDMS stamps. Thermal imprinting of the polymethyl methacrylate (PBMA) resist using PDMS nanohole template results in the formation of a nanocylinder array. The dimensions of this PBMA nanocylinder array, specifically its height (*H*_p_) and diameter (*D*_p_), could be modulated by varying the duration of the oxygen plasma etching process. This adjustment allowed for straightforward control of the adsorption area and, consequently, the resolution of the NP pattern. Atomic Force Microscopy (AFM) and SEM images revealed that the *H*_p_ of PBMA nanocylinders decreased from 310 ± 4.1 nm to 290 ± 5.3 nm, and further to 280 ± 6.7 nm as the plasma treatment duration increased from 40 s to 60 s, and finally to 80 s, respectively (**Figure** **2**a). Simultaneously, *D*_p_ decreased from 190 ± 7.6 nm to 130 ± 8.8 nm, and ultimately to 70 ± 10.8 nm (Figures S13 and S14, Supporting Information). The rate of decrease in *H*_p_ was comparatively slower than that of *D*_p_, which is attributed to the presence of a continuous layer of PBMA film layer beneath the nanocylinders. As etching occurs from the continuous PBMA film to the substrate surface, an array of PBMA nanocylinders with the underlying APTES or P4VP layers forms. By modulating the adsorption area, NP arrays with diameters ranging from 30 to 150 nm can be efficiently prepared. (Figure 2b; Figure S15, Supporting Information).

The interplay between the polymer pattern and AuNPs was fine‐tuned by regulating the ionic strength of the citrate‐capped AuNPs with NaCl salt under self‐limiting mechanism.^[^ ^52^, ^53^, ^54^ ^]^ In the case of a low NaCl concentration (e.g., 50 µm) for 60 nm AuNPs, over half of the adsorption sites were not filled (Figure 2c; Figure S16, Supporting Information). This was attributed to the large Debye length, which leads to significant repulsive interactions between the substrate surface and the migrating NP. Although high NaCl concentrations (e.g., 100, 200 µm) led to high filling ratios, most adsorption sites were occupied by multiple AuNPs. The increased ionic strength reduced the Debye length, leading to smaller interaction‐free energy barriers and allowing the NP to approach the positively charged circular template. Moreover, the small nearest interparticle separation allowed for accommodating multiple NPs on each circular adsorption site (Figure S17, Supporting Information). When the NaCl concentration was adjusted to ≈66 µm, a precise single AuNP array with high yield (99%) was obtained. The optimal assembly ionic strength conditions for other particle sizes are shown in the Table S2 (Supporting Information).

The AFM images and fast Fourier transform (FFT) patterns confirmed the high precision in positioning individual NPs within the periodic 600 nm (Figure 2d,e; Figure S18, Supporting Information). Furthermore, we analyzed the placement accuracy using an error statistics method based on computer vision technology (Figure S19a, Supporting Information). This method uses pixels as the smallest measurement unit and converts pixel values to length values in micrometer by normalization. By using multiple template matching and result filtering, the measurement error is minimized as much as possible (see Supplementary Text for details). An analysis of over 1000 NPs (each ≈120 nm in *D*_NP_) printed on silicon indicates a mean position offset down to 32 nm, surpassing those previously reported (Figure S19b, Supporting Information).^[^ ^45^, ^55^, ^56^, ^57^, ^58^, ^59^ ^]^ Additionally, for the smallest 30 nm particles, the position offset is ±11 nm, based on a statistical analysis of over 1000 particles (Figure 2f).

### 2.3 Consistent Plasmonic Response in NP Arrays

**Figure 3:**
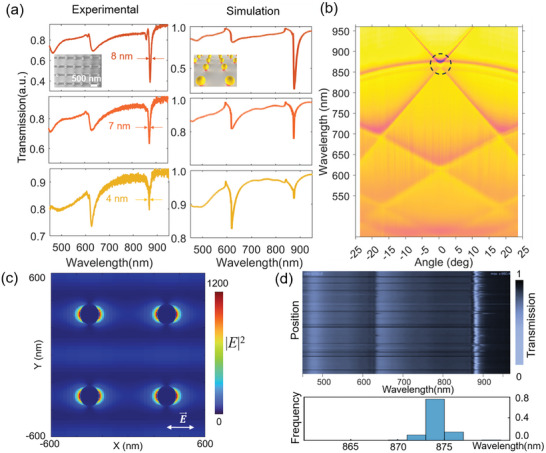
Optical properties of the NP arrays. a) Simulated and measured transmission spectra of Ag NP array *P* = 600 nm, *D*_NP_ = 150 nm (red line), *D*_NP_ = 125 nm (orange line), *D*_NP_ = 105 nm (yellow line) under normal incident light. The Ag NP array on a quartz substrate (n = 1.45) was immersed in DMSO (n = 1.47) for refractive index matching. b) Experimental dispersion diagrams of the Ag NP array, *D*_NP_ = 150 nm. c) Calculated near‐field distribution map of |*E*|^2^ in the X‐Y plane at 877.8 nm. d) Uniform spectral response in the Ag NP array immersed in DMSO. The waterfall plots display the transmission spectra continuously recorded from 100 collinear points at a step size of 50 µm.

The long‐range order and minimal position deviations of the NPs establish a robust foundation for a distinct and consistent SLR mode with a narrow spectral linewidth across the entire array. We utilized a polarization‐resolved momentum‐space measurement system to characterize the fabricated NP arrays s on quartz substrates (**Figure** **3**a). The refractive index was matched using dimethyl sulfoxide (DMSO), with a refractive index (n) of 1.47 on the quartz substrate (n = 1.45). We investigated the optical responses of square AgNPs arrays with a lattice parameter of *P* = 600 nm while varying diameter *D*_NP_ (Figure S20, Supporting Information). The array with *D*_NP_ = 105 nm exhibited a significantly stronger *Q* of 216 with a full width at half‐maximum (FWHM) of 4 nm at *λ* = 866.8 nm. To the best of our knowledge, these are among the highest *Q* values reported for colloidal NP arrays with the same lattice parameters and particle diameters.^[^ ^60^ ^]^ As the D_NP_ of Ag NP increased to 125 and 150 nm, the peak positions of SLR red‐shifted, and the *FWHM* broadened to 7 nm (*Q* = 124), and 8 nm (*Q* = 109), respectively. The phenomenon of *Q* decreasing with increasing *D*_NP_ can be explained by the formula as reported by Manjavacas and co‐workers: ^[^ ^61^ ^]^

(1) $$Q\approx {\left(P/D_{NP}\right)}^{9}$$

with the increase in *D_NP_*, the frequency (*f*) of SLR decreases (corresponding to a red shift in SLR wavelength) and the FWHM widens. This experiment trend aligns with our finite‐difference time‐domain (FDTD) simulation of transmission spectra and the formula proposed by Kravets and co‐workers:^[^ ^9^ ^]^

(2) $$f∼\omega_{p}-\frac{1}{16}\omega_{p}{D_{NP}}^{3}Re\left(S\right)$$

(3) $$\mathrm{FWHM}∼\frac{\omega_{P}{D_{NP}}^{3}}{\Omega }$$

where ω_*p*_ is the surface plasmon frequency, *S* is the retarded dipole sum and Ω is half‐width of surface plasmon resonance. Prominent transmission dips associated with SLRs can be found in both Ag and AuNPs arrays, which stem from the coupling between dipolar LSPRs of individual NPs and the zero‐order diffraction lattice modes. While maintaining the same SLR resonance intensity of 0.12, AuNPs of a *D*_NP_ of 120 nm exhibited a lower *Q* = 173 with FWHM = 5 nm compared with Ag NP arrays of 105 nm (Figure S21, Supporting Information). The Au NP arrays exhibit remarkable stability, as the FWHM of SLRs from these arrays remained unchanged after 1 year in ambient conditions (Figure S22, Supporting Information).

The angle‐resolved transmitted spectra illustrate the dispersion diagrams of the Ag NP arrays (Figure 3b; Figure S23, Supporting Information), where an evident band‐edge lattice mode can be found around **Γ** point corresponding to SLRs. The angle‐resolved results are consistent with the simulations results (Figure S24, Supporting Information). The incoming light is confined within the lattice plane, resulting in narrow linewidths and significantly amplified local electric fields at the NPs surface (Figure 3c).

Spatial reproducibility of optical response is crucial for the application of NP arrays. Therefore, we collected the transmission spectra on 100 consecutive regions with a constant 50‐µm step size on a 1 **×** 1 cm**^2^** Ag NP array sample (*P* = 600 nm, *D*_NP_ = 120 nm). Figure 3d displays an intuitive waterfall plot stitched together from the transmitted transmission spectra of 100 regions under refractive index matching conditions. A bright straight line, corresponding to the SLR transmittance at 873.5 nm, stands out against the background. This line is perpendicular to the lateral axis, which represents the consistency of the centre wavelengths and intensities of the SLR transmittance dips. The statistical results at the bottom of Figure 3d show the extracted peak, with the yield of SLR peaks concentrated ≈873.5 nm exceeded 72%.

### 2.4 Facile Integration and Hierarchical Patterning of NP Arrays

**Figure 4:**
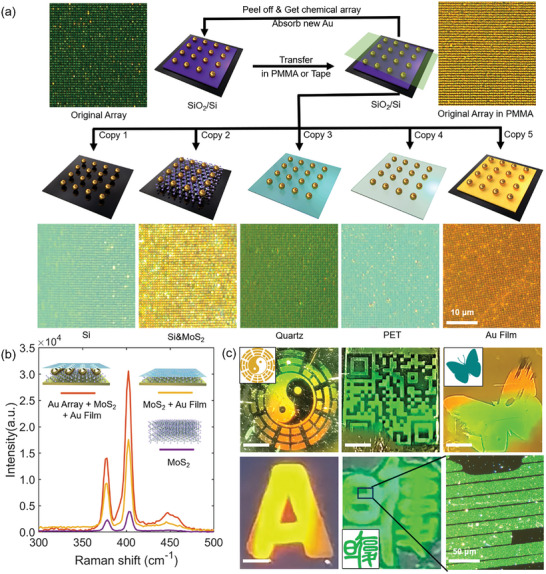
AuNPs arrays transferred onto various substrates and hierarchically patterned with intricate designs. a) Dark‐field images of AuNPs arrays on SiO_2_/Si, can be replicated multiple times and transferred onto other substrates with PMMA as the transfer media. b) Representative SERS spectra recorded from MoS_2_ samples on different substrates. c) Photographs of NP hierarchical patterns with intricate designs. The secondary structures on the scales of micrometer and millimeter were created using photolithography. The rightmost photo shows an enlarged dark‐field image of the logo of Fudan University. Scale bar: 0.3 cm.

Centimeter‐scale NP arrays with single‐particle spatial ordering can be readily prepared on various substrates by selecting appropriate linkers for surface modification and NP immobilization (Figure S25, Supporting Information). However, when integrating NP arrays into functional devices, operating procedures such as heating, plasma etching, and solvent washing may introduce contamination and damage to the active surfaces, leading to performance degradation. An effective solution is the clean transfer of NP arrays from the original substrates to the target ones. As shown in **Figure** **4**a, high‐quality AuNPs arrays produced by our renewable chemical pattern on a SiO_2_/Si substrate after multiple copy cycles can be faithfully transferred with high yield (≈99%) to various kinds of substrates. Such as silicon (a semiconductor), silicon dioxide (an insulator), molybdenum disulfide (MoS_2_) monolayer (2D material), quartz (optical glass), PET film (flexible polymer), and gold film (metal). It is worth noting that this facile and fast transfer printing was mediated by the Scotch transparent tape. Proper adhesion of the tape allows NP arrays to be completely detached from the original substrates and strongly adhere to the target surfaces. The whole process circumvents the use of solvents, high temperature, sacrificial layers, and other surface treatment, thereby minimizing the risk of undesired changes to surface properties. These features are crucial for seamless integration into functional devices. As a proof of concept, we attach a tape‐supported AuNPs array onto a MoS_2_ monolayer on an Au film, creating a nanoparticle‐on‐mirror (NPoM) structure. This NPoM structure supports plasmonic gap modes. Nanogaps can trap light in small volumes and produce enhanced electromagnetic fields which give rise to advanced optical phenomena, for example, SERS. Here, the sandwiched MoS_2_ monolayer is characterized using SERS (Figure 4b). Two representative vibration peaks at 378 and 405 cm⁻¹ are well preserved, corresponding to the in‐plane vibrations of Mo and S atoms (*E*_12_ _g_) and the out‐of‐plane lattice vibration of S atoms (*A*_1_ _g_).^[^ ^62^ ^]^ The intensity of these two characteristic peaks shows a two‐fold enhancement as compared to the bare MoS_2_ monolayer on an Au film. It is thus clear that the optically transparent Scotch tape brings no harm or interference to the optical properties of the MoS_2_ monolayer. Moreover, the NPoM structure with an ordered AuNPs array shows a larger enhancement. This enhancement is attributed to the effective confinement of incident light within the nanocavity, exciting a highly localized plasmonic mode with a strong electric field intensity. Consequently, this greatly enhances the lattice vibrations of the analyte MoS₂, confirming the effectiveness of the MoS₂ spaced NPoM hybrid structure and suggesting its potential for future SERS detection.^[^ ^63^ ^]^

The chemical pattern NP transfer strategy is also effective for creating complex patterns, such as letters, Bagua Array, QR code, and logos, with high resolution and precision (Figure 4c; Figure S26, Supporting Information). Coupled with its recyclable nature, this approach offers new insights into “roll‐to‐roll” processing. Some of the devices can be patterned directly, the NP solution serves as the “ink” and the customized polymer pattern prepared on a flexible substrate acts as the “stamp” (Figure S27, Supporting Information). Moreover, the first layer of particles adsorbed on the substrate can be modified and used as adsorption sites for a second layer. This second layer can also be transferred using tape while maintaining the integrity of the first layer (Figure S28, Supporting Information). This continuous printing method enables the creation of intricate designs and patterns using NPs, opening up new possibilities in various scientific and technological applications such as electronics, photonics, catalysis, and biosensing.

## 3 Conclusion

In conclusion, we have developed a precise, scalable and recyclable method for the fabrication of NP patterns that combines soft thermal nanoimprinting and chemical assembly. This approach enables the creation of well‐defined nanoparticle arrays with single‐particle resolution and yields exceeding 99%. Our approach allows for the fabrication of high‐resolution patterns from NPs ranging in size from 30 to 150 nm, which can be transferred onto different substrates. Our technique has demonstrated exceptional repeatability, with the capability of generating NP arrays consistently for >60 cycles while maintaining a high yield of ≈95%. Furthermore, this method facilitates the integration of these arrays into complex systems, achieving precise pattern transfers with uniformity over large areas (up to 1 cm^2^) and positional accuracy of ±11 nm for 30 nm NPs. This capability is crucial for creating intricate, multicomponent, and nonplanar structures that are difficult to fabricate using conventional lithographic techniques. This work provides a versatile platform for the scalable and reproducible fabrication of nanoparticle patterns, paving the way for advancements in multiple scientific and technological domains including electronics, photonics, catalysis, and biosensing.

## 4 Experimental Section

### Materials

Sodium citrate tribasic dihydrate (ACS reagent, ≥99.0%), L‐ascorbic acid (ACS reagent, ≥99.0%), APTES (99%), P4VP (M_w_ ≈160 000), PBMA (Mw ≈70 000), PMMA (Mw ≈350 000), and were purchased from Sigma–Aldrich. Gold(III) chloride tetrahydrate (HAuCl_4_·4H2O, ≥99.9%), AgNO_3_ (≥99.8%), NaCl (≥99.5%), NH_3_·H_2_O solution (25‐28%), toluene (≥99.5%), absolute ethanol (≥99.7%) and acetone (≥99.5%), isopropanol (≥99.7%) were purchased from Sinopharm Chemical Reagent. PDMS SYLGARD 184 Silicone Elastomer Kit was purchased from Dow.The relief nanopatterned silicon masters, consisting of square and hexagonal arrays of posts with diameters of 250 and 190 nm, a height of 350 nm, and a pitch of 600 nm, were purchased from Max Levy Autograph, Inc. All chemicals and reagents were used as received. The deionized water was purified using a Millipore water purification system with a minimum resistivity of 18.2 MΩ·cm.

### Substrate Preparation

Substrates of silicon, silicon dioxide, gold‐plated silicon, and fused quartz (15 mm × 15 mm) underwent a cleaning process in acetone, isopropanol, ethanol, and water sequentially before transfer printing. Similarly, flexible PET samples (250 µm thick), both plain and gold‐plated (500 nm), were cleaned in isopropanol prior to use.

The preparation of MoS_2_ on a substrate was achieved through the exfoliation of natural MoS_2_, utilizing ultraviolet‐release tape. The tape with MoS_2_ was contacted with the substrate and heated on a 130 °C hotplate for 2 min. After removing from the hotplate, the tape was carefully peeled off.

### Chemical Modification of Substrate

Silicon, silicon dioxide and fused quartz substrates (15 mm × 15 mm) were cleaned using oxygen plasma at 60 mtorr, 35 s.c.c.m., and 60 W for a duration of 5 min. The substrates were immersed in a 0.2% v/v APTES/ethanol solution for 30 min, followed by a 3 min ultrasonic bath and gentle drying with N_2_. Subsequently, they were heated at 120 °C for 3 h. For P4VP modification, the gold‐plated silicon, cleaned using oxygen plasma, underwent a 30 min immersion in a 1% v/v P4VP/ethanol solution.

### Gold Nanoparticle Synthesis

AuNPs of varying diameters were synthesized using a seed‐mediated growth method by Ziegler et al. with slight modifications.^[^ ^64^ ^]^ Gold seeds were produced via the citrate reduction method, involving the addition of 1.21 mL HAuCl_4_ (1 wt%) to 99 mL DI water in a two‐neck flask, heated and stirred until boiling. Post‐injection of 4 mL sodium citrate (1 wt%), heating was ceased once the solution turned red.

The gold seeds were grown to desired diameters by adding 12 mL seed solution and 132 mL DI water to a three‐neck flask. 8 mL each of precursor (0.2 wt% HAuCl_4_) and reductant (0.25 wt% sodium citrate and 0.5 wt% ascorbic acid) solutions were simultaneously injected using a two‐channel syringe pump, followed by heating for 30 min to yield first‐generation (G1) AuNPs with diameters of 30 nm.

G1 AuNPs were further grown to second‐generation (G2) AuNPs. To obtain 60 nm G2 AuNPs: starting with 30 mL of G1 AuNPs, add 4 mL of precursor and 4 mL of reductant solutions. To obtain 80 nm G2 AuNPs: using 22 mL G1 AuNPs, add 8 mL of precursor and 8 mL of reductant solutions. To obtain 100 nm G2 AuNPs: using 12 mL of G1 AuNPs, adding 8 mL of precursor and 8 mL of reductant solutions. For AuNPs of 120 nm, a third growth step was employed using 12 mL gold seeds, 15 mL G1, and 110 mL G2. The diameters were confirmed via TEM images (Figure S2, Supporting Information). The AuNPs were then purified and redispersed in various NaCl solutions. Add hydrochloric acid to achieve the desired pH. The concentration of AuNPs solution was estimated by the method provided by Haiss et al.^[^ ^65^ ^]^

### Silver Nanoparticles Synthesis

AgNPs were synthesized using a seeded growth method developed by Xing and coworkers.^[^ ^66^ ^]^ Initially, silver seeds were prepared by mixing 1 mL of sodium citrate solution (1 wt%), 0.25 mL of AgNO_3_ solution (1 wt%), 0.2 mL of NaCl solution (20 mm), and 1.05 mL of DI water, followed by stirring for 5 min. After adding 80 µL of ascorbic acid to 47.5 mL of boiling water, the mixture was injected and boiled for 1 h. Upon cooling, bright yellow silver seed sols were obtained. To synthesize 150 nm AgNPs, 6 mL of the silver seed sol was concentrated to 1 mL via centrifugation. Subsequently, 200 µL(350 uL for 125 nm AgNPs and 400 uL for 105 nm NPs) of the concentrated Ag seed sol was added to 9.46 mL of DI water in a 20 mL glass vial under vigorous stirring at room temperature. Sequentially, 140 µL of silver‐ammonia complex (Ag[NH_3_]_2_OH) aqueous solution and 4 mL of ascorbic acid aqueous solution (2.5 mM) were added. The UV–vis extinction spectrum exhibited a dipolar plasmon peak centered at 601 nm for 150 nm AgNPs, 557 nm for 125 nm AgNPs and 490 nm for 105 nm NPs (Figure S20, Supporting Information). The resulting AgNPs dispersion was separated through centrifugation and redispersed in a NaCl solution. Add hydrochloric acid to achieve the desired pH. The concentration of AgNPs solution was estimated by the method provided by Paramelle et al.^[^ ^67^ ^]^

### PDMS Mold Fabrication

Silicon masters with relief nanopatterns were passivated with a fluorosilane (tridecafluoro‐1,1,2,2‐tetrahydrooctyl‐1‐trichlorosilane) for 24 h in a vacuum desiccator to inhibit adhesion to the PDMS template. Following passivation, a coating of GELEST‐hPDMS was applied to the fluorinated mold. GELEST‐hPDMS(1:1 base to curing agent ratio) was spin‐coated onto the fluorinated mold at 3000 rpm for 60 s and thermocuring for 10 min at 60 °C. Then SYLGARD 184‐PDMS (10:1 base to curing agent ratio) was poured on the masters and cured for 2 h at 70 °C. Upon completion of the curing process, the PDMS was carefully peeled off from the master and step revealed the array topographical mold.

### Patterning of Chemisorbed Site on Substrate

The silicon wafer, modified with APTMS or P4VP, was coated with a 13 mg mL^−1^ PBMA/toluene solution by spin‐coating at 3000 rpm and 3000 rpm s^−1^ acceleration for 40 s. The resulting PDMS molds were placed in contact with the wafer and secured with a pressure clamp. Post solvent evaporation at 100 °C for 25 min, the PBMA in the wells caused PDMS mold swelling. Upon mold removal, PBMA nanocylinders were formed on the wafer. These cylinders were patterned in the 70–190 nm range using oxygen plasma at 60 mtorr, 35 s.c.c.m., and 60 W for a duration of 40–80 s. Subsequently, the wafer was subjected to an ultrasonic treatment in acetone for 1 min and air‐dried and patterned APTES SAMs or P4VP array were obtained. For more intricate patterning, a layer of photoresist was applied to the wafer via spin‐coating at 3000 rpm for 60 s. The desired pattern was then etched using a laser direct writing lithography machine. Post a 1 min oxygen plasma cleaning, the patterned wafer underwent a final cleaning process with lye developer.

### Iterative Replication and Transfer of Nanoparticle Array

The cycle began with the directed assembly of AuNPs arrays. Substrates patterned with APTES SAMs or P4VP were incubated in a solution of citrate‐stabilized AuNPs for 3 h to ensure full absorption. The resulting periodic AuNPs arrays were then transferred using commercially available 3 m 600 Scotch tape or PMMA. The tape was gently attached to the prepared array surface, torn off, and transferred to other substrate surfaces. The stripped array substrate was recycled and could be reused in the same or another nanoparticle solution. PMMA films were spin‐coated onto the nanoparticle arrays using a 300 µL PMMA/toluene solution (500 mg·mL^−1^) at a rotational speed and acceleration of 3000 rpm for 60 s. The PMMA‐covered substrate was then immersed in a photolithography development solution for 5 min, after which the PMMA film was peeled off. The PMMA film was transferred to other substrates with the assistance of water and air drying, allowing the stripped substrates to be put into subsequent use.

### FDTD Simulation Details

The optical and electric properties of plasmonic structures were simulated using the FDTD method via commercial software. The Broadband Fixed Angle Source Technique (BFAST) in FDTD was employed to simulate the angle‐resolved transmittance spectra. The dielectric functions for gold were sourced from Johnson and Christy's 1972 values. To ensure accurate electric and magnetic field calculations within the nanoparticles, a uniform mesh size of 2 nm was utilized. Periodic boundary conditions were established along the X and Y directions, with 20 perfectly matched layers applied along the Z direction. A plane wave light source, with a wavelength range of 400−900 nm, was injected along the Z axis at normal incidence. A frequency domain power monitor was positioned on the substrate's opposite side to record transmittance data. The frequency points were set at 250 for arrays with an air superstrate and 750 for a glycerol superstrate, due to the extremely sharp spectra features resulting from SLR.

### Characterization

Microscopic structures were imaged using a Gemini Ultra55 (Zeiss) SEM and a HT7800 (Hitachi) TEM. The morphology of PBMA and AuNPs arrays, as well as the thickness of polymer films, were characterized using a Dimension Fastscan (Bruker) AFM. The Zetasizer Nano ZS90 (Malvern Instruments) analyzer and a SevenCompact (METTLER TOLEDO) pH meter were used to measure the zeta potential and pH of the plasmonic nanosphere sols, respectively. XPS tests were conducted with an ESCALAB Xi+ (Thermo Fisher Scientific, Al Kα radiation, hv = 1486.6 eV). The UV–vis extinction spectra of plasmonic NP dispersions and quartz substrate‐supported plasmonic AuNPs array were collected using a Cary 60 UV–vis spectrophotometer (Agilent). The Raman spectra were recorded using a Renishaw/NT‐MDT (In Via Qontor /NTEGRA Spectra II) spectrometer with a 532 nm laser. Dark‐field microspectroscopy imaging were captured with a Leica DMi8C/HRS‐300 CCD hyperspectral imaging system. Angle‐resolved transmitted spectra were obtained using home‐made extinction spectroscopy. By employing an objective lens, the optical Fourier transform and acquired the angle‐resolved transmissive spectra were performed on the back focal plane with a spectrometer.

## Conflict of Interest

The authors declare no conflict of interest.

## Supporting information



Supporting Information

## Data Availability

The data that support the findings of this study are available from the corresponding author upon reasonable request.
