# Supplementary material for: Sustainable Fabrication and Transfer of High‐Precision Nanoparticle Arrays Using Recyclable Chemical Pattern Templates
Source: Adv Sci (Weinh). 2024 Dec 7;12(5):2407393. doi: 10.1002/advs.202407393 (PMC11791935; doi:10.1002/advs.202407393)
Supplement: Supplementary file 1 — Supporting Information [file ADVS-12-2407393-s001.docx]

Supporting Information

**Sustainable Fabrication and Transfer of High-Precision Nanoparticle Arrays Using Recyclable Chemical Pattern Templates**

*Huaining Zha, Wenjie Zhang, Peng Chen, Jing Tao, Li Qiu, Fan Yang, Shunsheng Ye *, Yutao Sang*, and Zhihong Nie**

**Supplementary Figures**


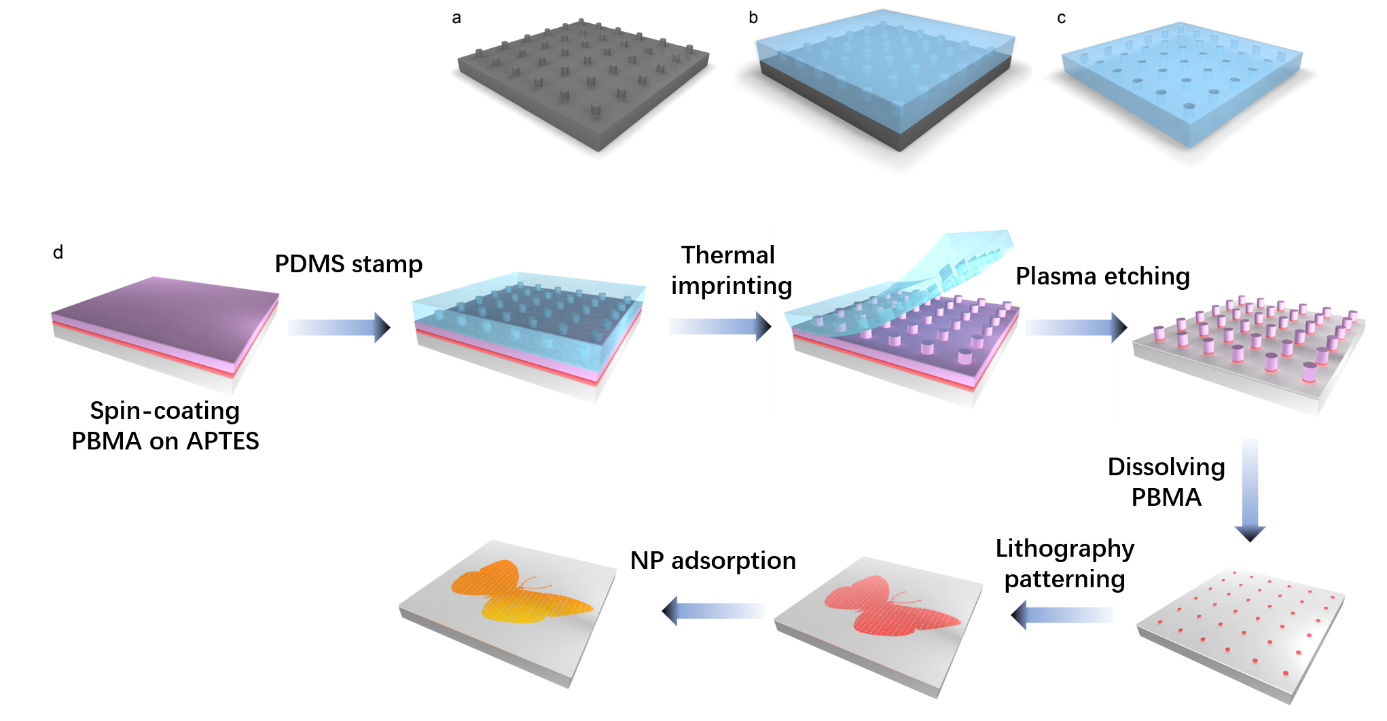


**Figure S1.** Schematic illustration of the nanofabrication process of large-area chemical pattern array. (a, b, c) Schematic illustration of the mold turning process utilizing a nanocylinder square array silicon template for the creation of a Polydimethylsiloxane (PDMS) stamp. (d) Schematic illustration of fabrication of patterned NP array by thermal nanoimprinting. In the fabrication of intricate patterns, we begin by spin-coating a photoresist solution onto a substrate that already features a lattice of chemical patterns. Next, the desired pattern is etched into the substrate using a laser direct writing lithography machine. Following this, the lye developer is applied and subsequently cleaned by oxygen plasma. Finally, the surface chemical pattern lattice of the final complex pattern is obtained through nitrogen blowing. The resulting complex pattern exhibits an accuracy of up to 20 micrometers or smaller.


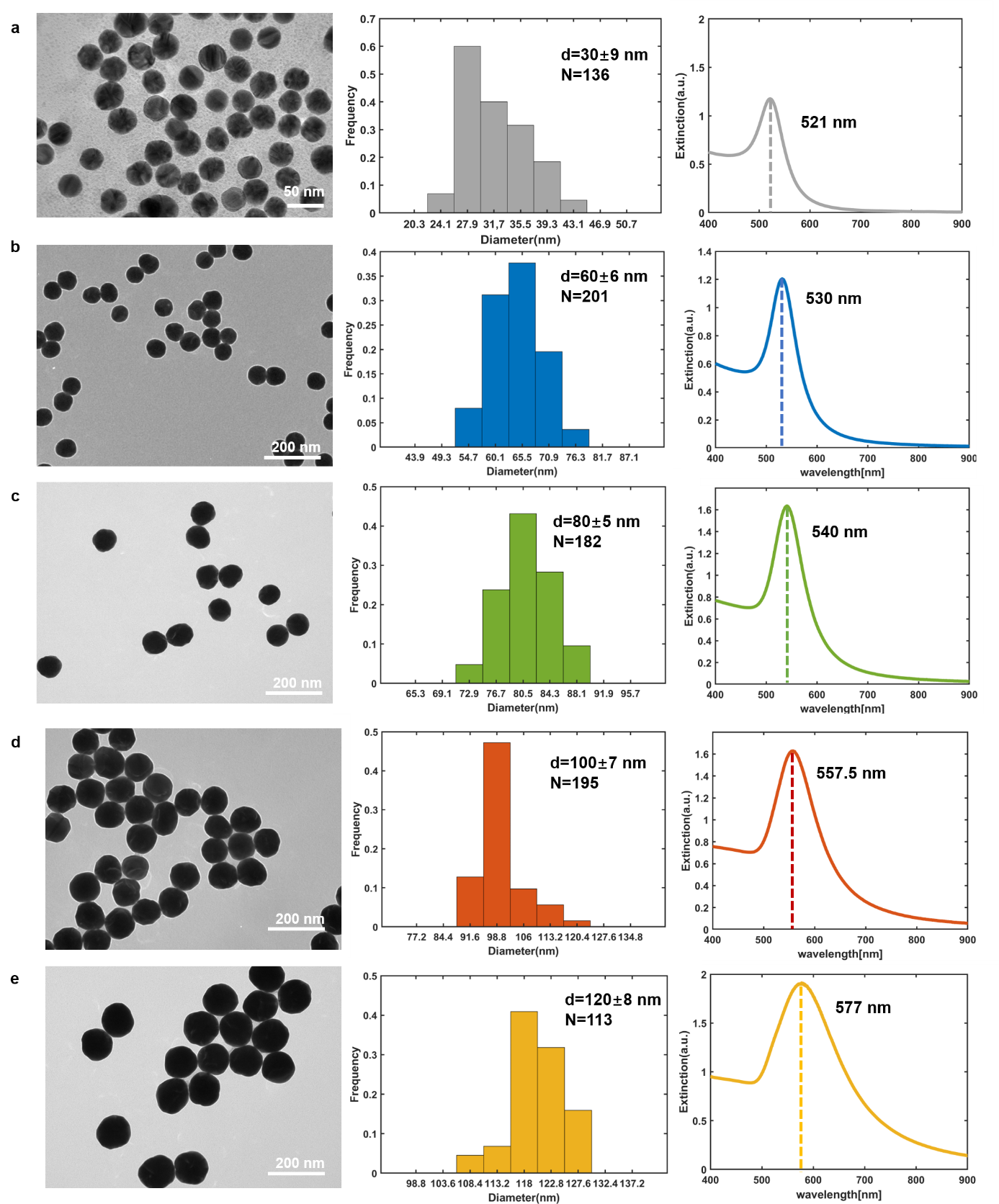


**Figure S2.** Characterizations of synthetic AuNPs properties. (a–e) Representative TEM images, corresponding particle size distribution, and UV–vis absorption spectra of as-synthesized. (a) AuNPs with 30 ± 9 nm diameters, (b) AuNPs with 60 ± 6 nm diameters. (c) AuNPs with 80 ± 5 nm diameters, (d) AuNPs with 100 ± 7 nm diameters. (e) AuNPs with 120 ± 8 nm diameters.

**Adsorption Efficiency** In our method, the colloidal concentration was in large excess. For instance, the colloidal concentration of 100 nm Au NP is approximately *C_NP_* ≈1.4 × 10⁻^11^ mol/L. In our experiment, we typically immersed the substrate in a 20 mL NP solution. The number of NPs in a 20 mL solution is calculated as: *n* = *C_NP_* × *V* × *N_A_* = 1.69 × 10^11^. Each use consumes approximately 2.32 × 10^8^ NPs for a 1 cm x 1 cm array with a period of 600 nm. Therefore, we have a massive excess of colloidal NPs.


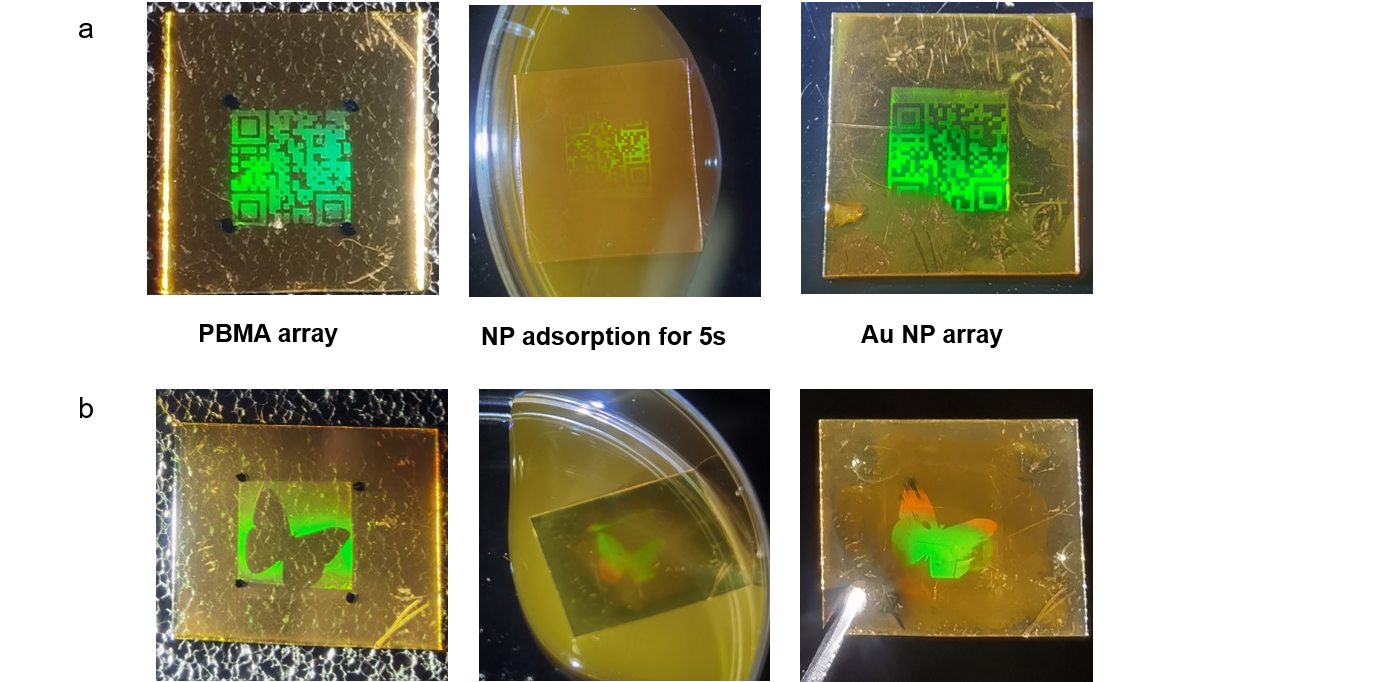


**Figure S3.** Photographs of preparation process of patterned arrays in the form of a QR code (a) and a butterfly (b). The transition from the PBMA array to the AuNPs array is illustrated, with adsorption achievable in just five seconds.


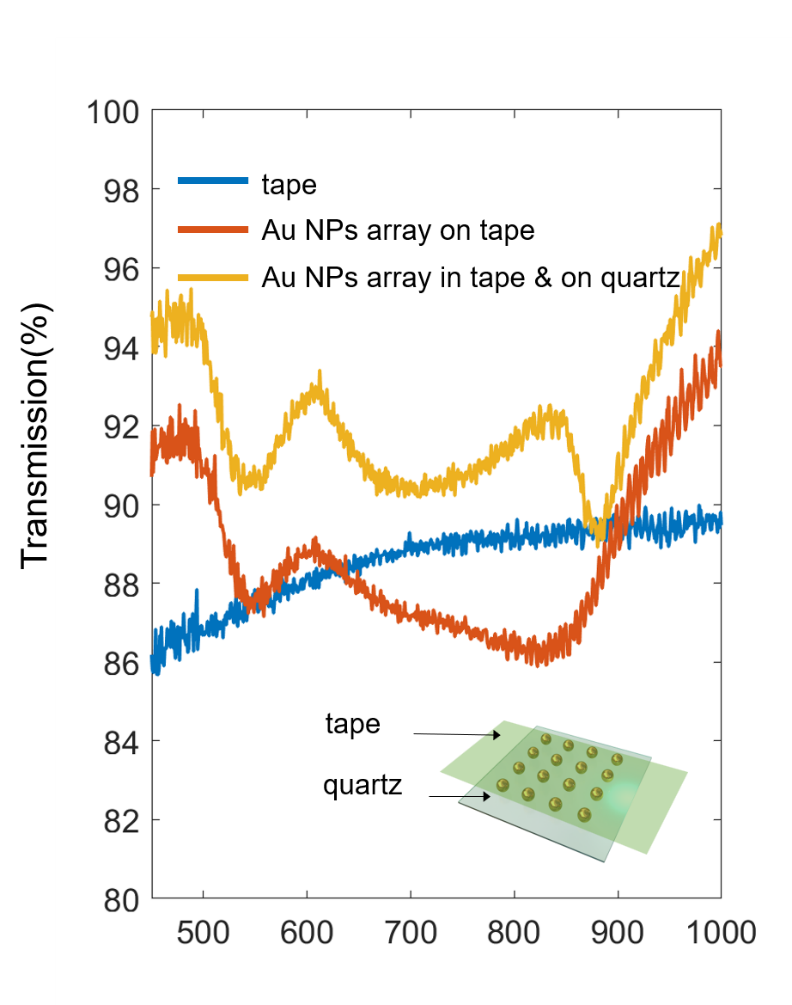


**Figure S4.** Experimental measured transmission spectra of 120 nm AuNPs array. The backing material of the Scotch® 600 tape is unplasticized polyvinyl chloride (UPVC). The refractive index (n) of UPVC is 1.54, which roughly matches the refractive index of the quartz substrate (n=1.45). This refractive index matching explains why the SLR peak at 875 nm is present once the tape is immobilized again on quartz. When one side of the NP is in contact with tape and the other side with air (n=1), the SLR peak disappears owing to the refractive index mismatch.


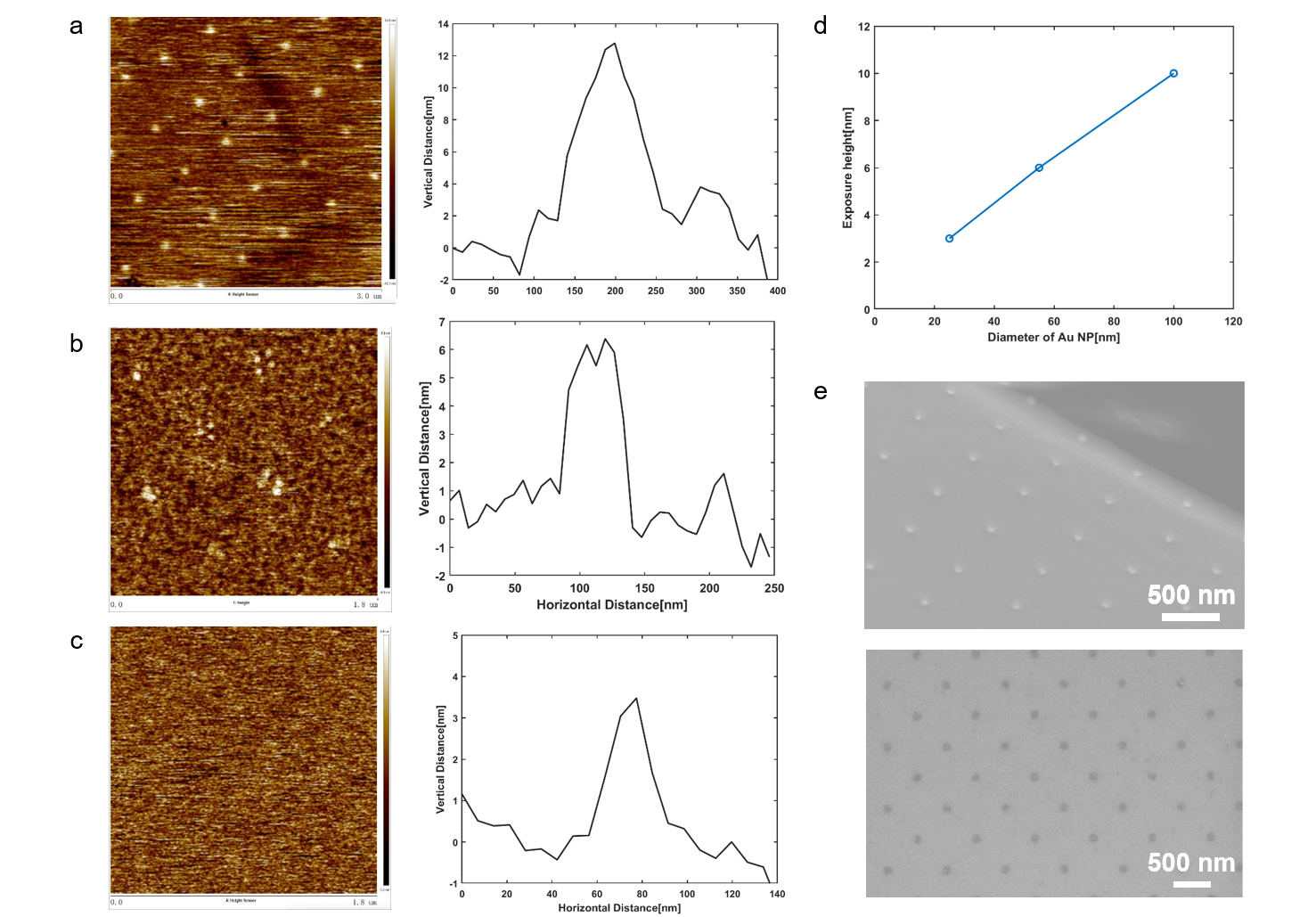


**Figure S5.** Characterization of AuNPs arrays transferred onto PMMA. (a-c) AFM images and corresponding height profiles of AuNPs transferred onto PMMA with the height profile indicating particle protrusion above the PMMA film surface. (d) The exposure height as a function of diameter of AuNPs. (e) SEM image of 100 nm AuNPs array transferred onto PMMA (top) and corresponding silicon surface after transfer (bottom).


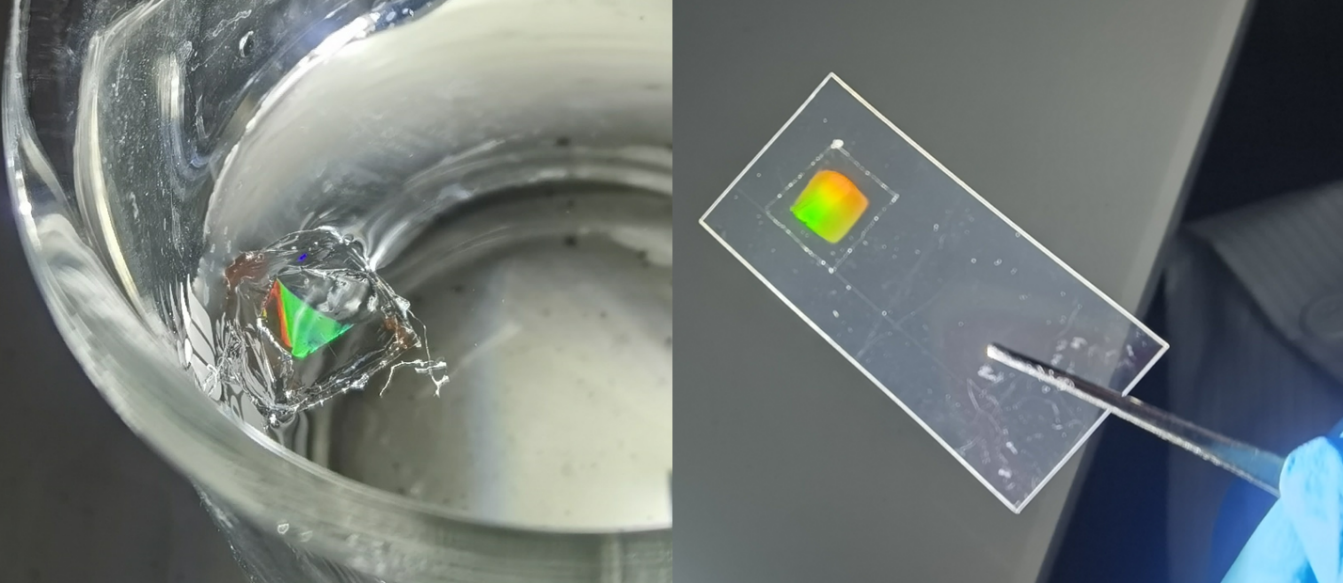


**Figure S6.** Photograph of the PMMA film attached to the target surface with the aid of water.


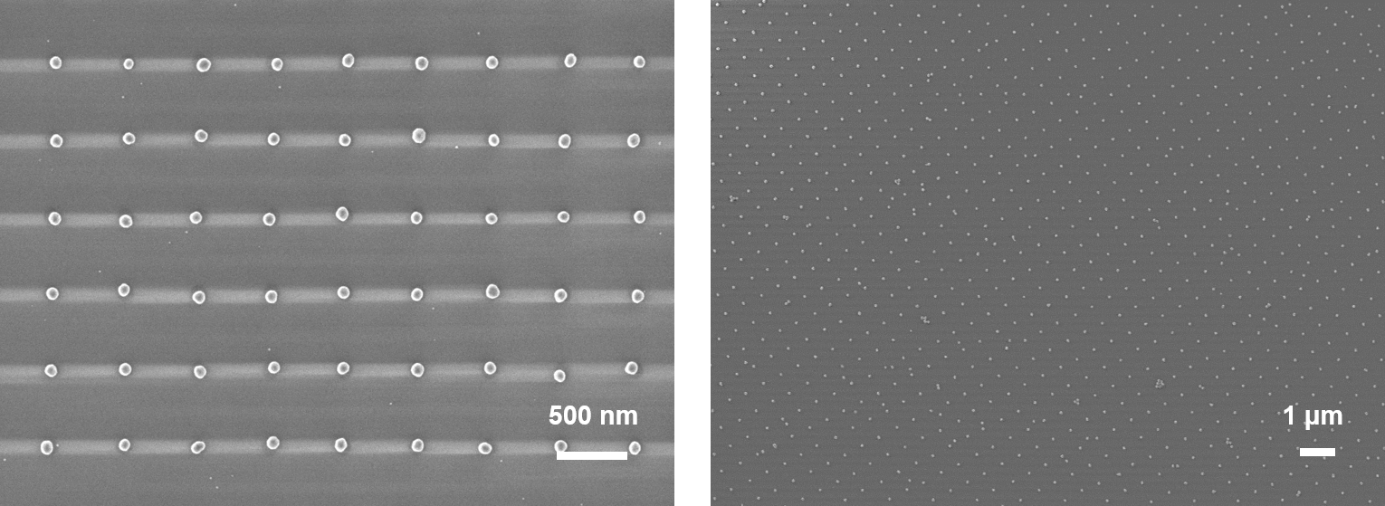


**Figure S7.** SEM images of the AuNPs array adsorbed on chemical pattern array which has been subjected to repeated transfer processes for 60 cycles.


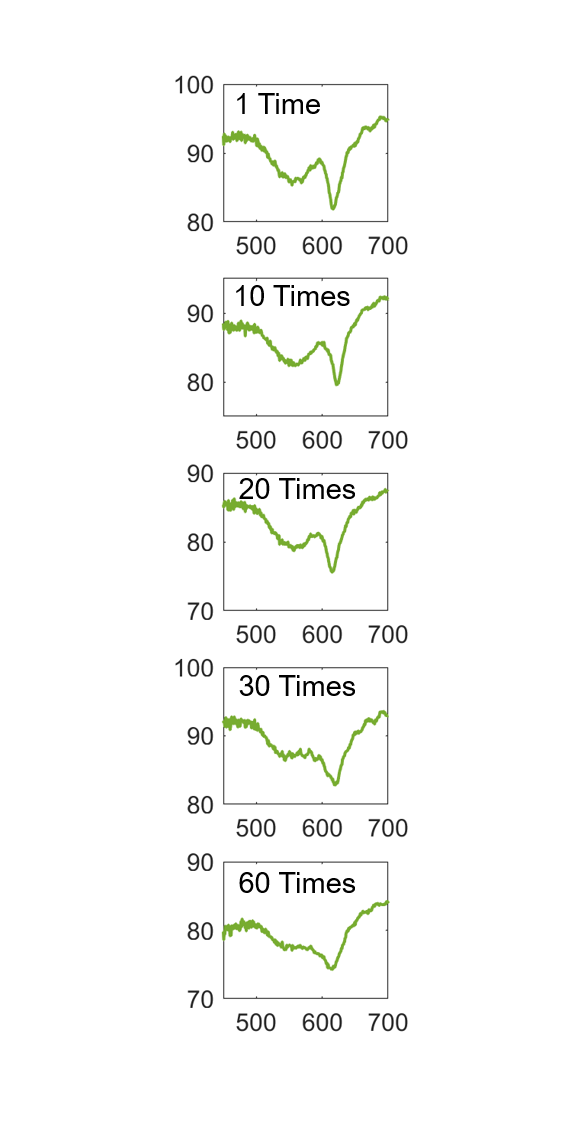


**Figure S8.** Transmission spectra of new NP transferred onto PMMA when the refractive index matches that of the transferred quartz substrate, corresponding to the result in Figure 1e.


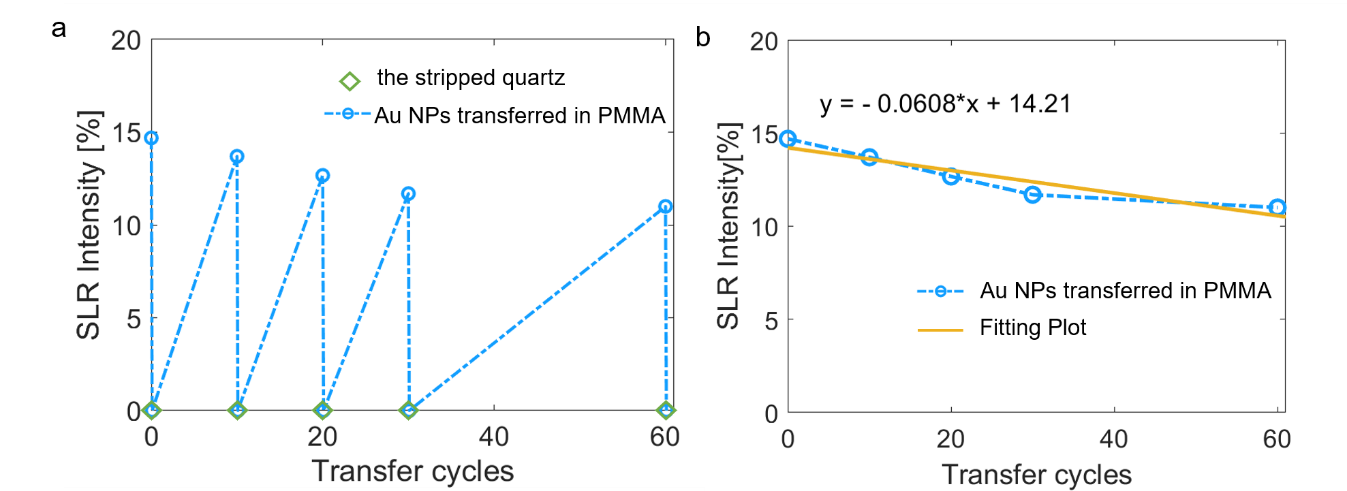


**Figure S9.** Variation in the intensity of the SLR peak across transfer cycles. (a) Transmission intensity of new NP transferred onto PMMA with different cycles. (b) Following a linear function fitting of the transmission intensity, it is anticipated that the 533rd surface plasmon resonance (SLR) transmission peak will vanish.


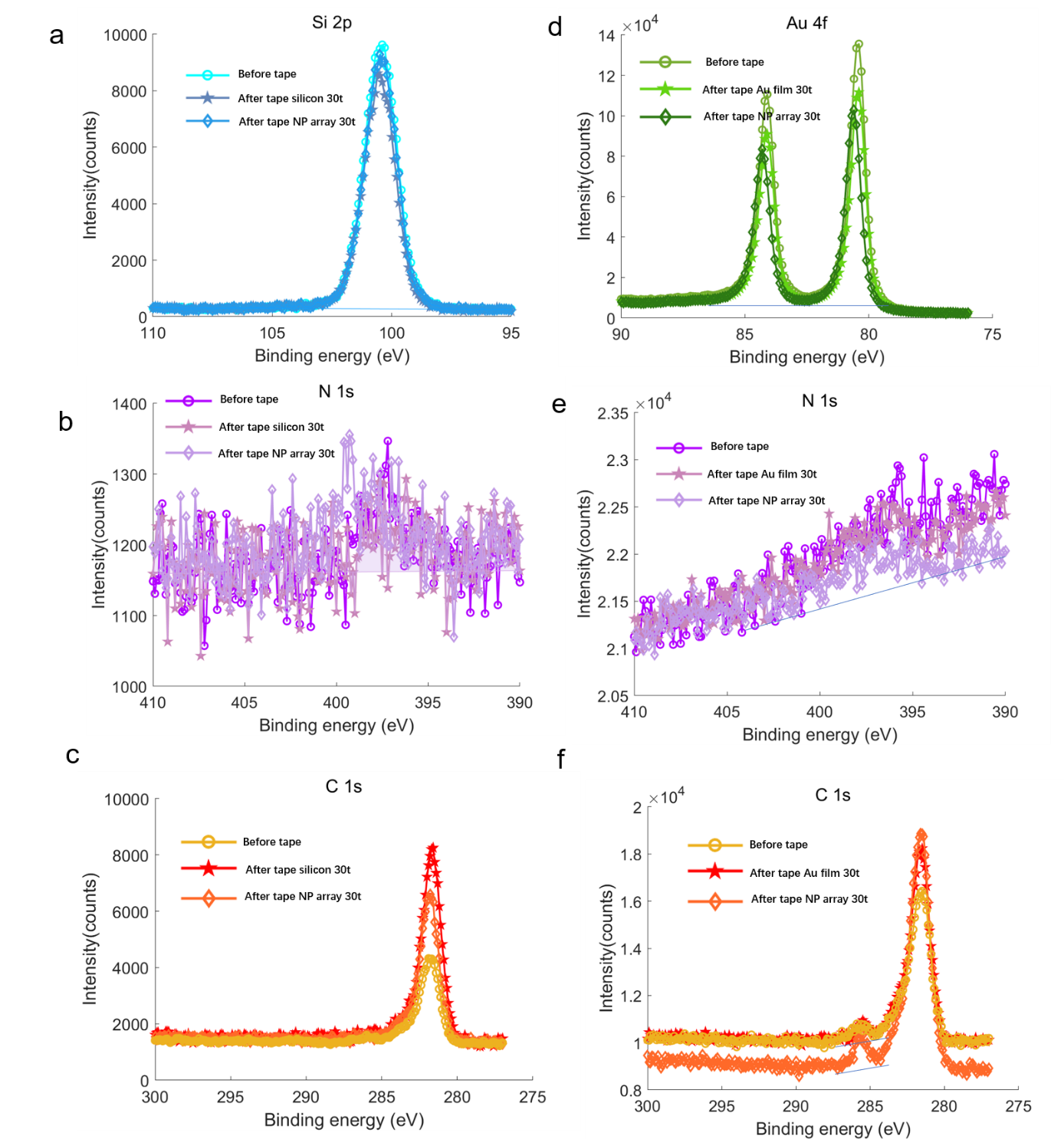


**Figure S10.** The high-resolution XPS spectra of (a-c) APTES on SiO_2_/Si and (d-f) P4VP on Au film presented after the nanoparticle array was transferred 30 times with tape and taped 30 times directly on the chemical pattern array. These are compared with the initial substrate with the chemical pattern array.


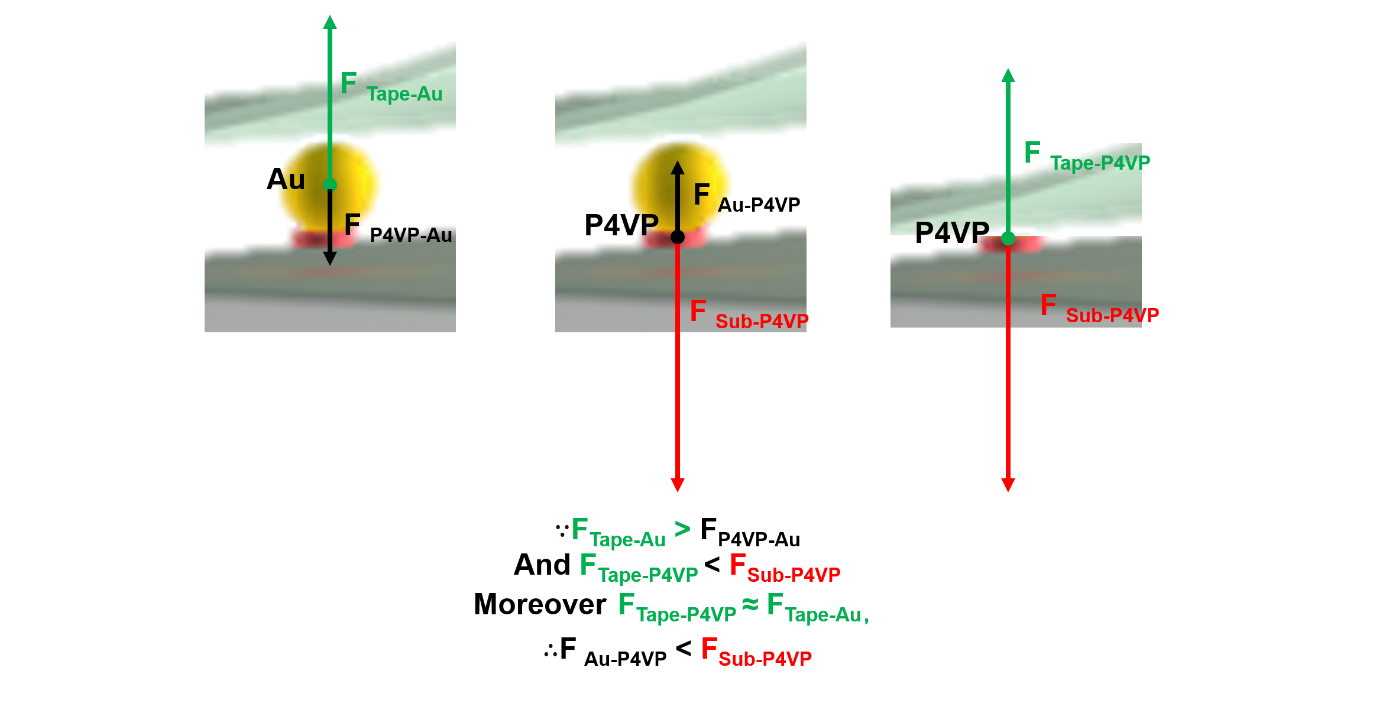


**Figure S11.** The mechanics model of ‘tape-AuNPs-P4VP-substrate’. Indeed, the force analysis reveals that if P4VP remains adhered to the substrate when the tape directly contacts it, the AuNPs will not take away the P4VP. In other words, the P4VP remains secure even when the tape is attached to the gold nanoparticles.

**The direct adsorption of AuNPs onto the fully P4VP-modified substrate indirectly reflects changes in P4VP density.** Considering that P4VP is a polymer, the situation becomes more nuanced. In the “point contact” scenario with the gold nanoparticles, the adhesive forces may not be sufficient to retain the P4VP. However, when in “surface contact” with the gold substrate, the P4VP has a more stable interaction. In practice, we observe a delicate dance: some P4VP molecules may indeed be carried away by the gold nanoparticles, albeit in small quantities. The interplay between these forces—adhesion, surface area, and material properties—shapes the intricate behavior of this system.


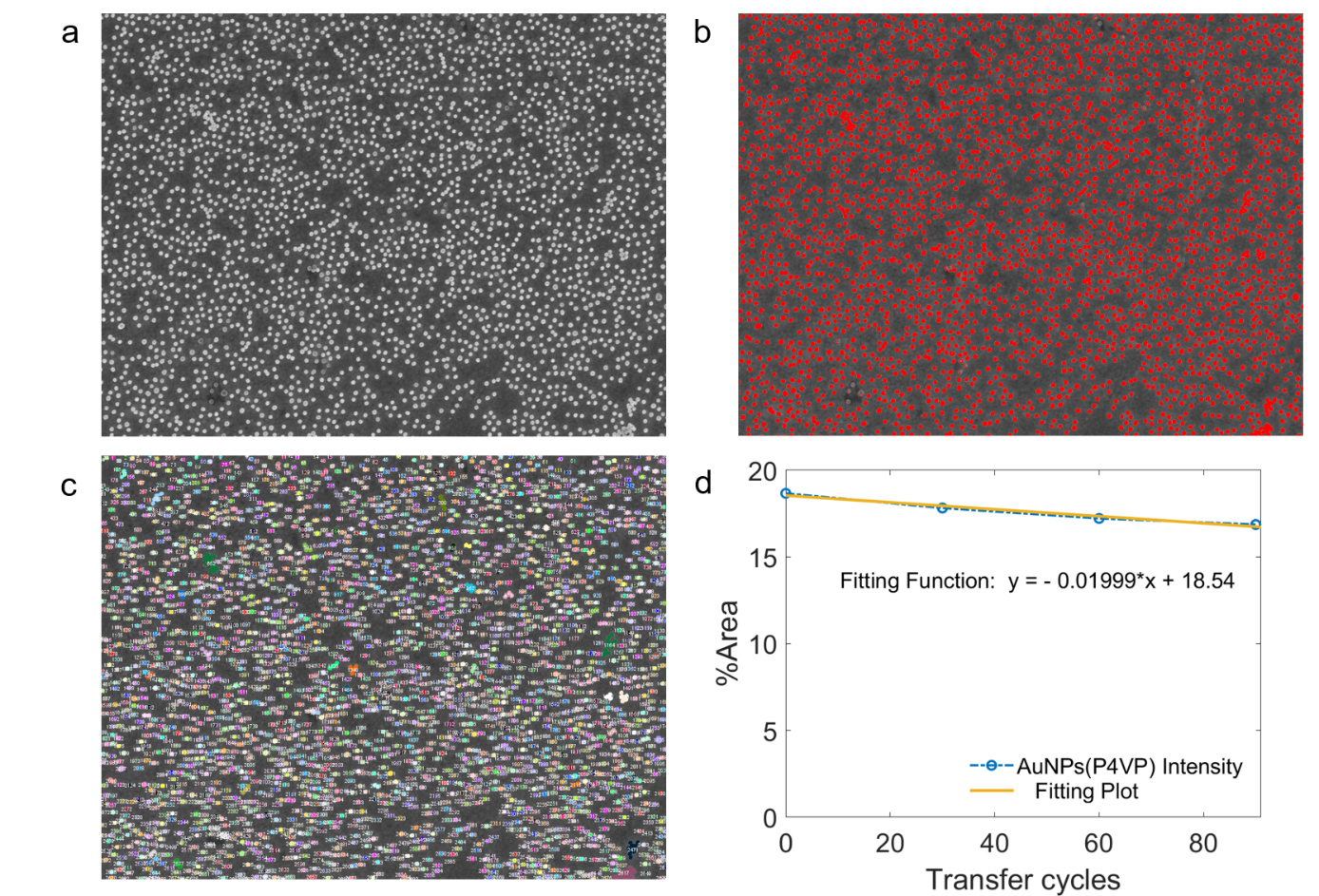


**Figure S12.** After pasting P4VP 30 times, 60 times and 90 times with tape, the particle density of gold nanoparticles adsorbed on P4VP was analyzed in five regions at 7.5um*1um. (a) SEM Images. (b) “Color threshold” image. (c) Particle analyze with overlay masks. (d) Function fitting of the particle density across transfer cycles.


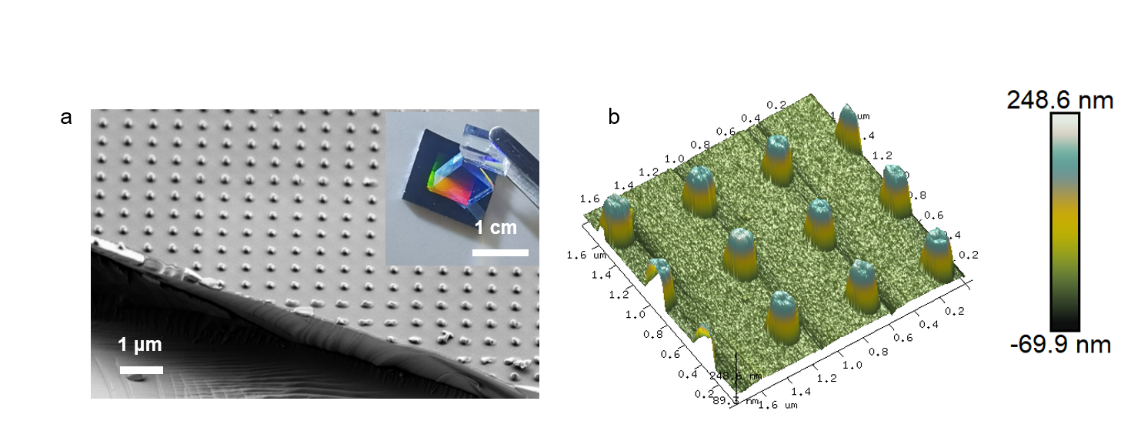


**Figure S13.** Characterization of PBMA nanocylinder arrays. SEM image (a) and tapping-mode 3D (b) AFM image of a PBMA nanocylinder array fabricated by soft lithography and thermal nanoimprinting.


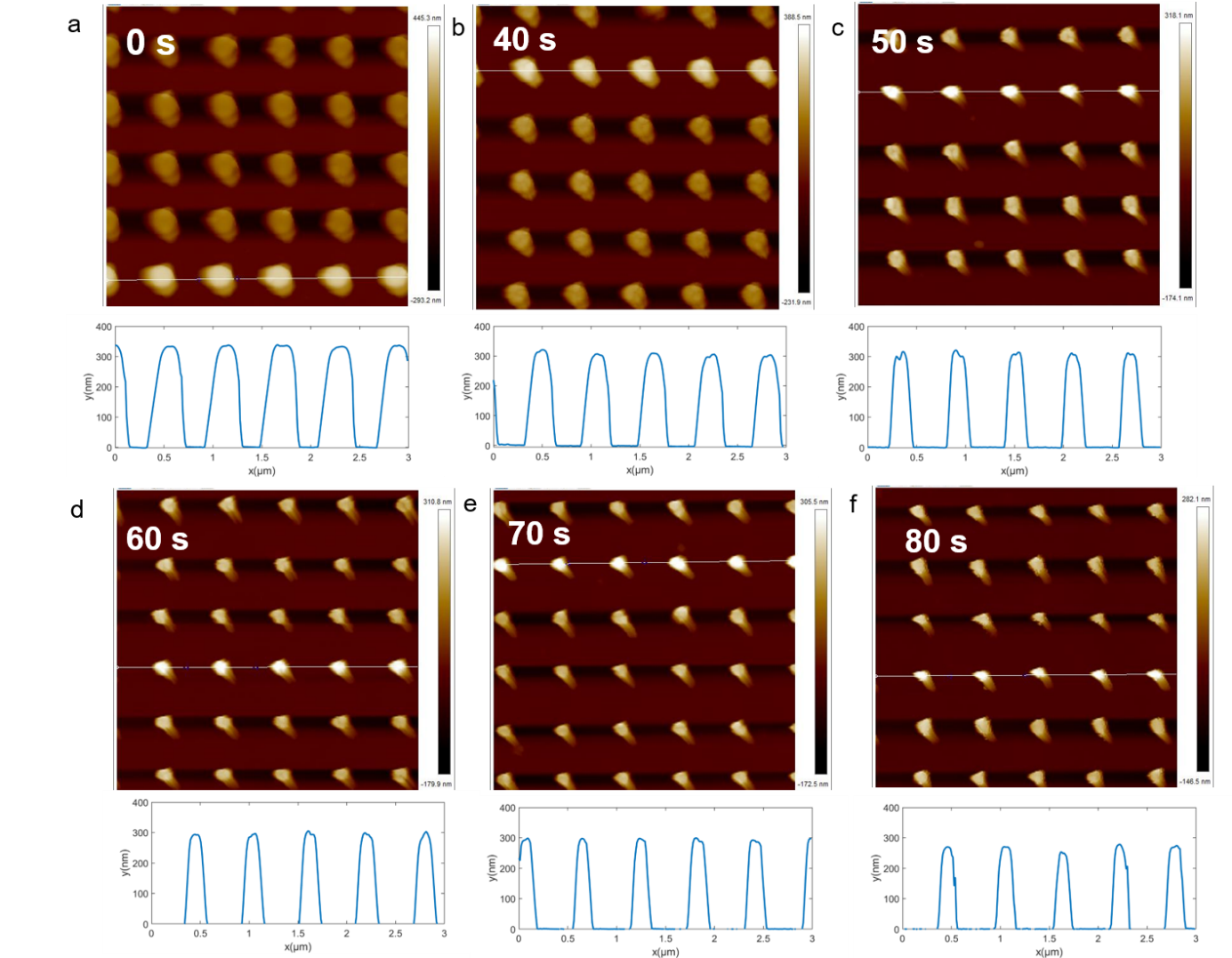


**Figure S14.** Tapping-mode AFM images and corresponding height profiles of PBMA nanocylinder arrays obtained at different O_2_ plasma etching times: 0 s (a), 40 s (b), 50 s (c), 60 s (d), 70 s (e) and 80 s (f).


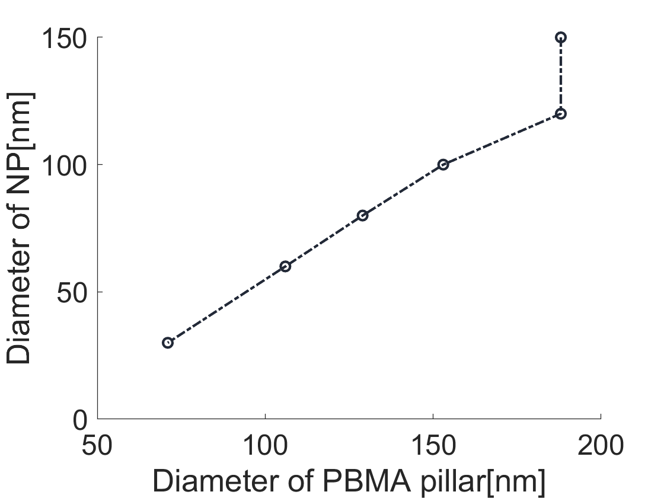


**Figure S15.** The diameter of PBMA nanocylinders is adjusted by the diameter of NPs.


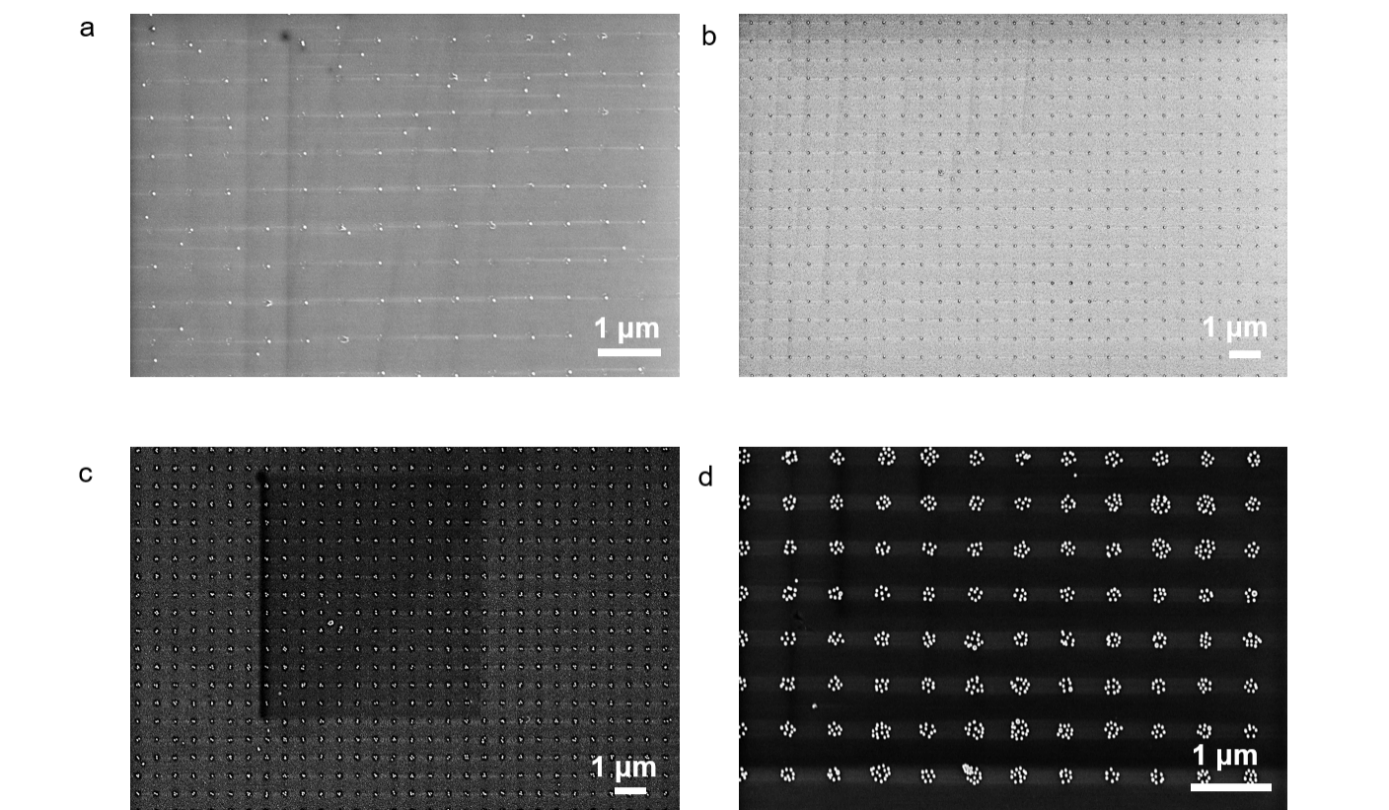


**Figure S16.** Characterization of AuNPs arrays under various salt concentration adsorption conditions. (a-d) SEM images of AuNPs arrays under normalized salt concentrations of 50 mM (a), 66 mM (b), 100 mM (c), and 200 mM (d).

**Self-limiting** **principle** Once a NP is absorbed on the chemical circular template, the electrostatic landscape changes, preventing other NPs from approaching the template. This results in self-limiting single-particle placement, as shown in Figure S17. Self-limiting single-particle placement can be realized when two conditions are met. First, the initial NP should experience minimal resistance (i.e., weak upward forces and a small free energy barrier) as it approaches the chemical template. Second, once the first NP is in place, subsequent NPs should encounter significant upward forces or a large free energy barrier, preventing their approach to the chemical template. High NaCl concentrations may reduce the Debye length and interaction free energy barriers, allowing multiple NPs to approach the substrate


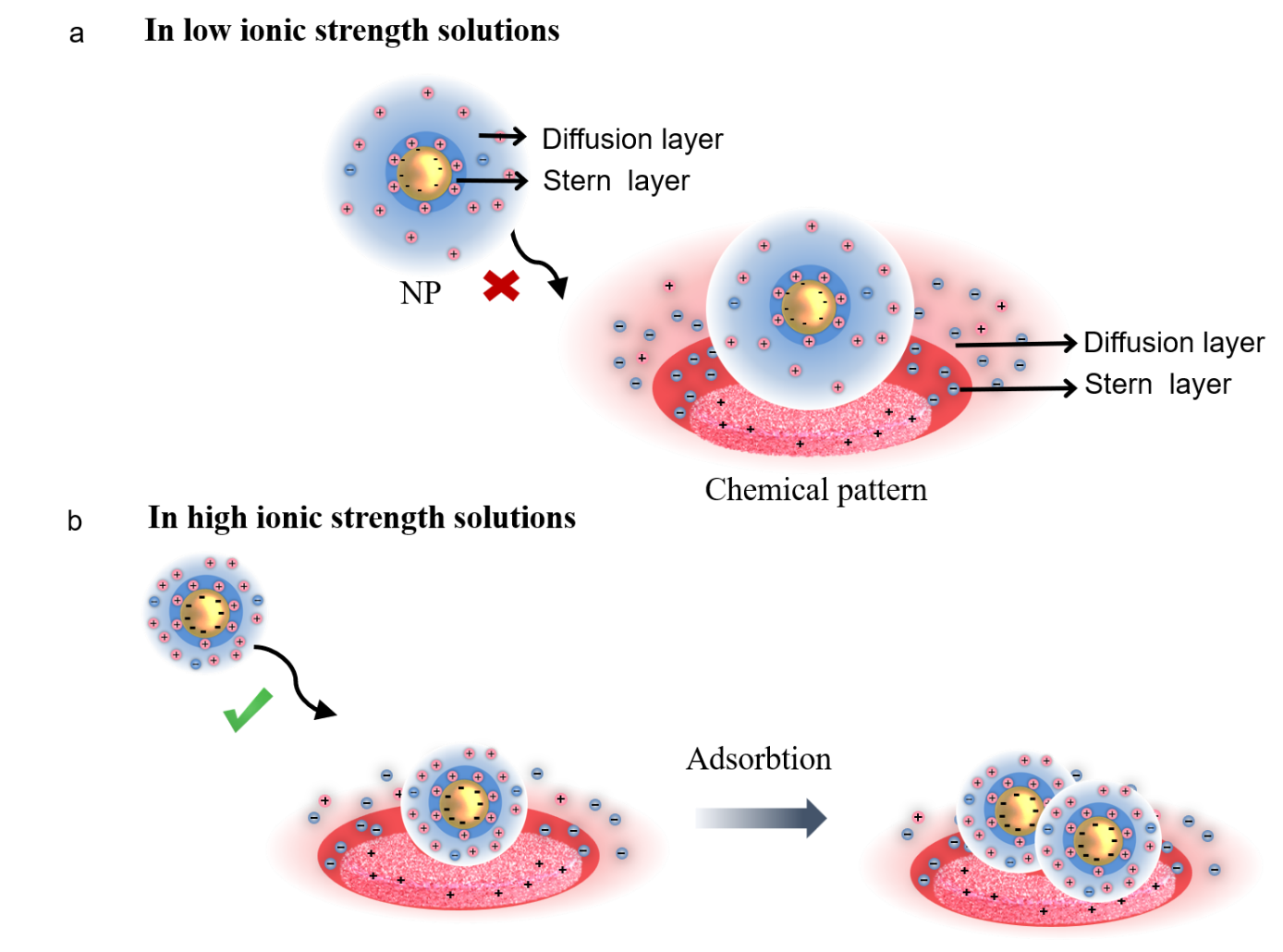


**Figure S17.** Schematic drawings of the formation of NP under self-limiting mechanism in low ionic strength solutions (a) and in high ionic strength solutions (b).


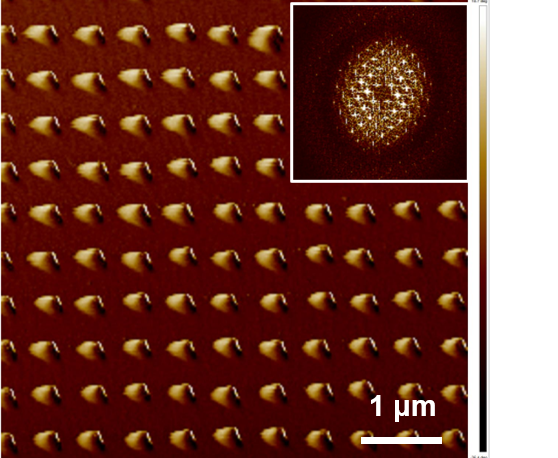


**Figure S18.** AFM images and the corresponding FFT patterns of 100 nm AuNPs tetragonal arrays.

**
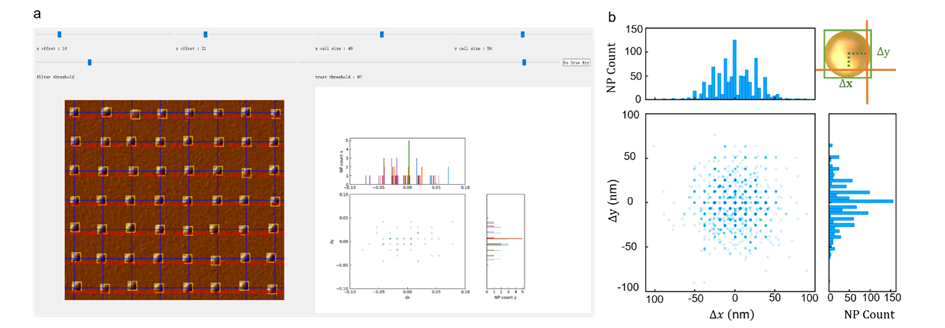
**

**Figure S19.** The error statistics method based on computer vision technology. (a) Analysis of placement accuracy utilizing an error statistics method based on computer vision technology. (b) Distribution of individual 120 nm NPs lateral offset relative to their designated position as defined by the template. The data, collected from 1,000 nanoparticles, indicates a mean placement accuracy of ± 32 nm post-chemisorption (80% numbers).


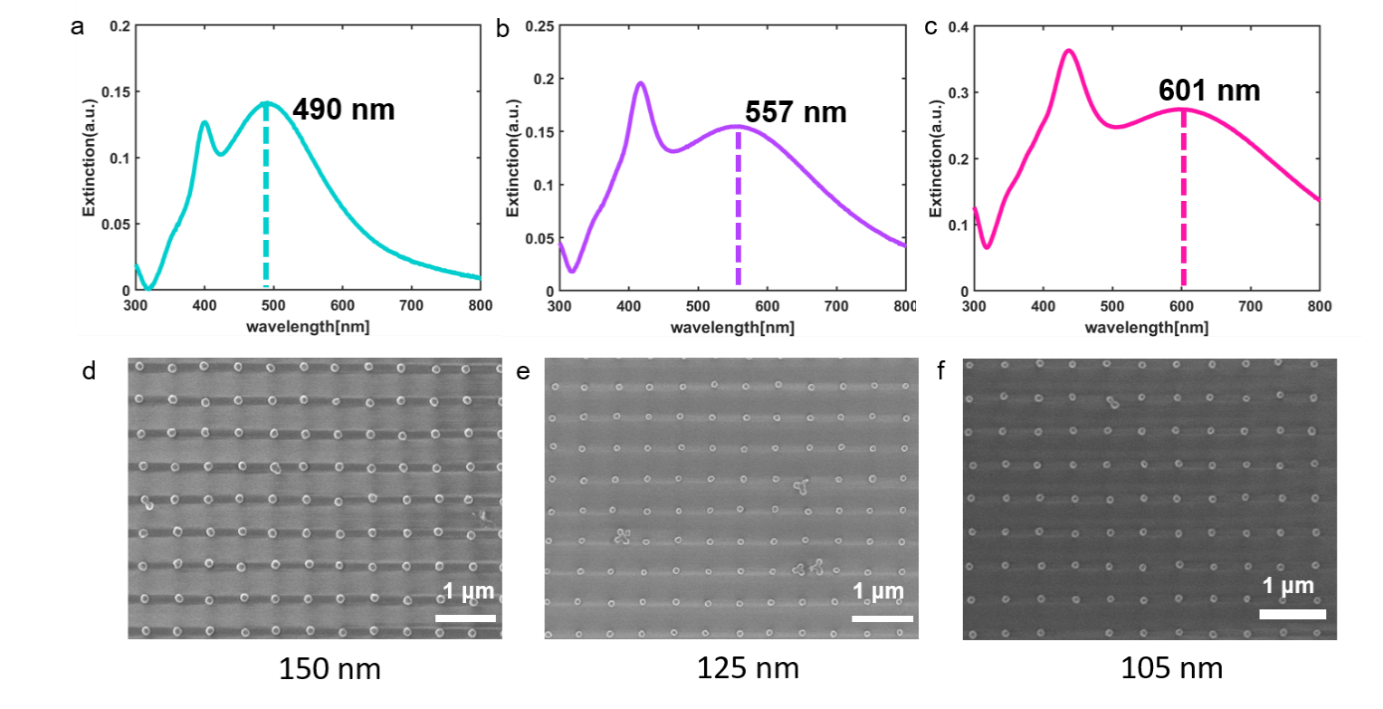


**Figure S20.** UV–vis absorption spectra and SEM images of synthetic Ag NPs properties. (a, d) AgNPs with 150 ± 9 nm diameters. (b, e) AgNPs with 125 ± 6 nm diameters. (c, f) AgNPs with 105 ± 5 nm diameters.


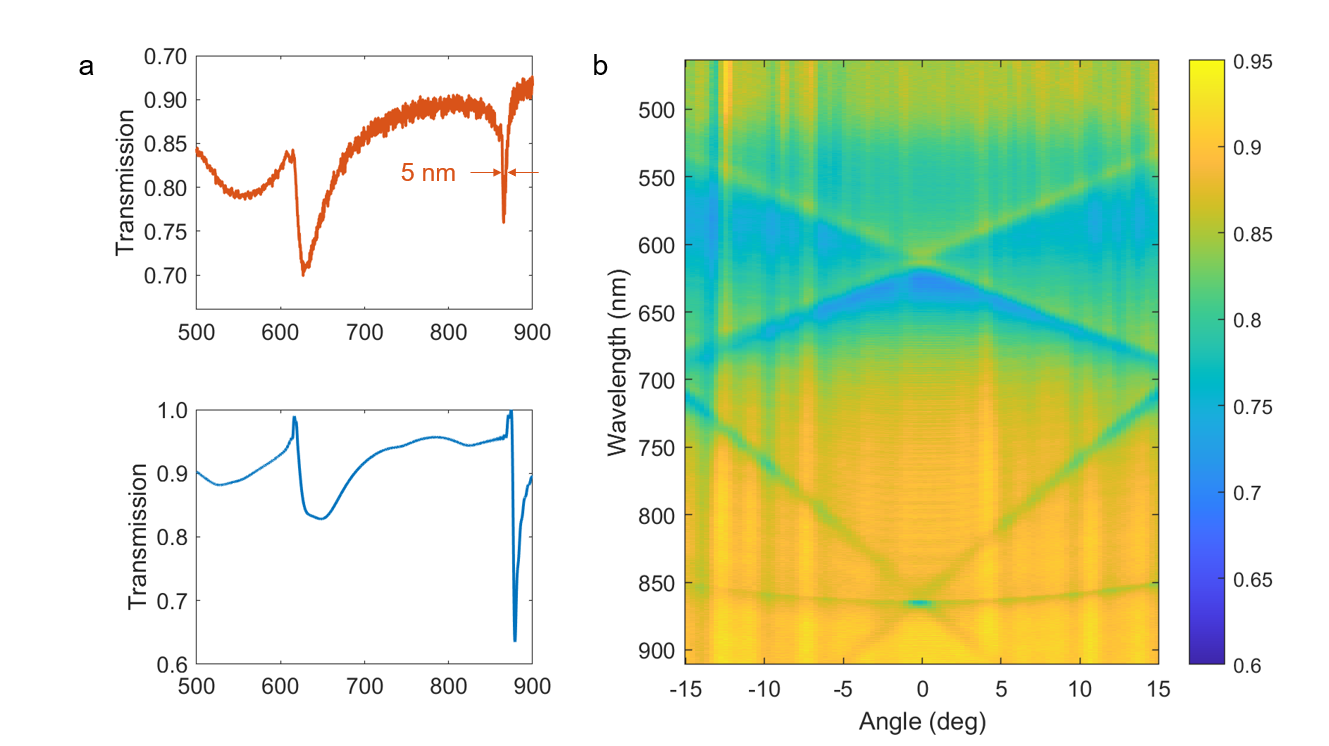


**Figure S21.** Optical properties of 120nm AuNPs array. (a) Simulated and experimental measured transmission spectra of Ag NP array under linear polarized light. (b) Experimental dispersion diagrams of AuNPs arrays under TE polarization.


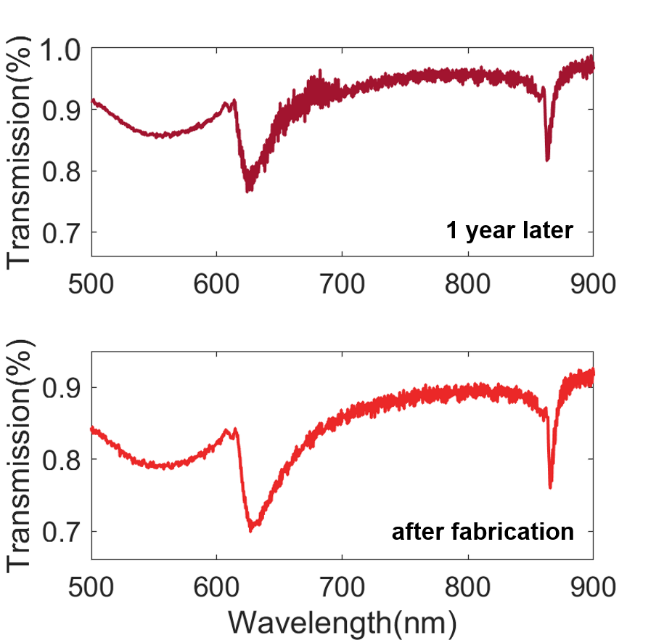


**Figure S22.** Transmission spectra of 120 nm Au NP array after 1 year (top) and after fabrication (bottom).


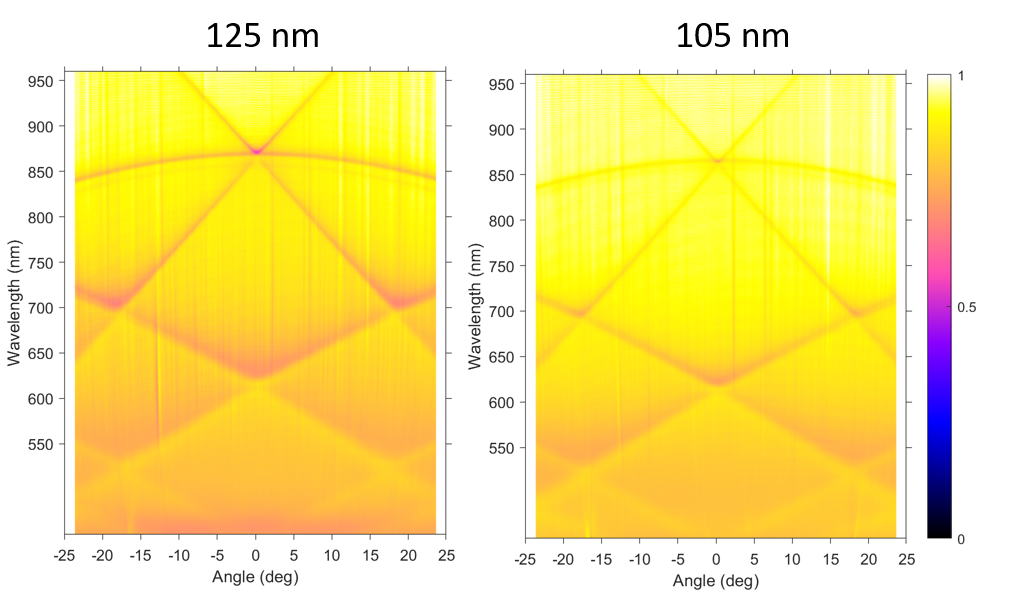


**Figure S23.** Optical characterization results of 125nm and 105nm Ag NP array.


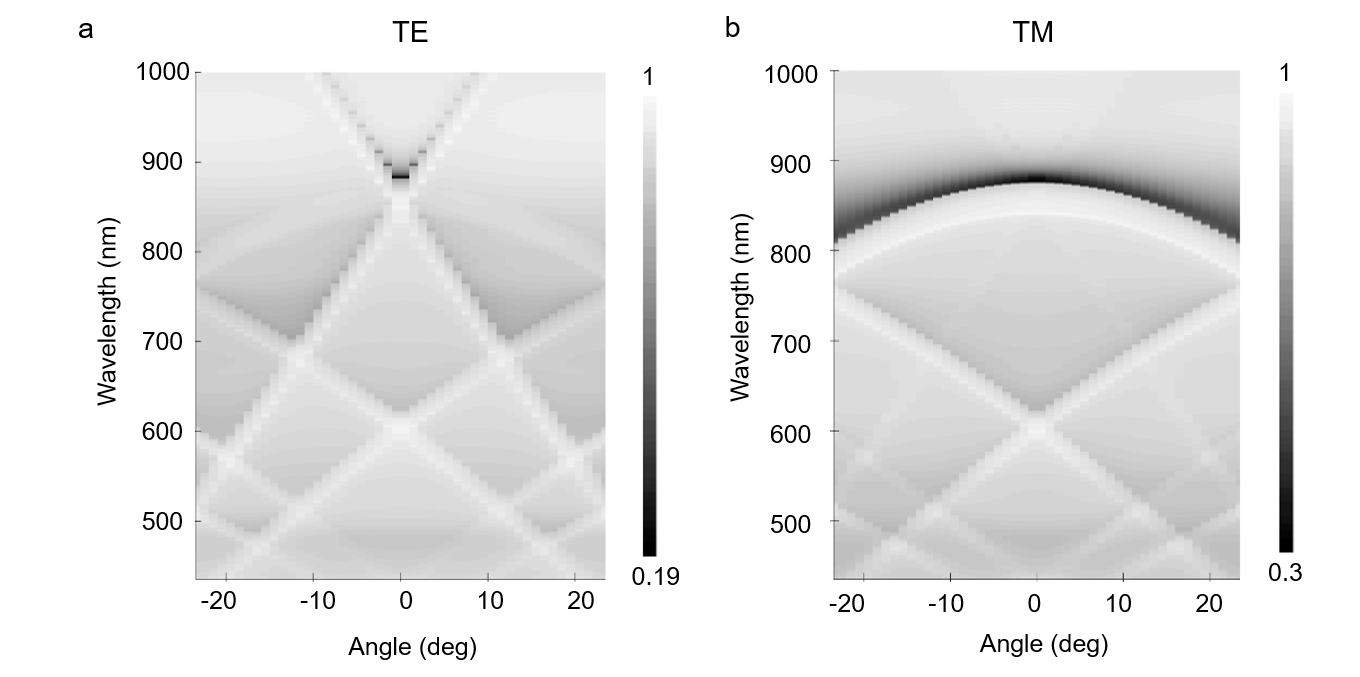


**Figure S24.** Simulated dispersion diagrams of Ag NP arrays (*P* = 600 nm, *D*_NP_ = 150 nm) under TE (a) and TM (b) polarization


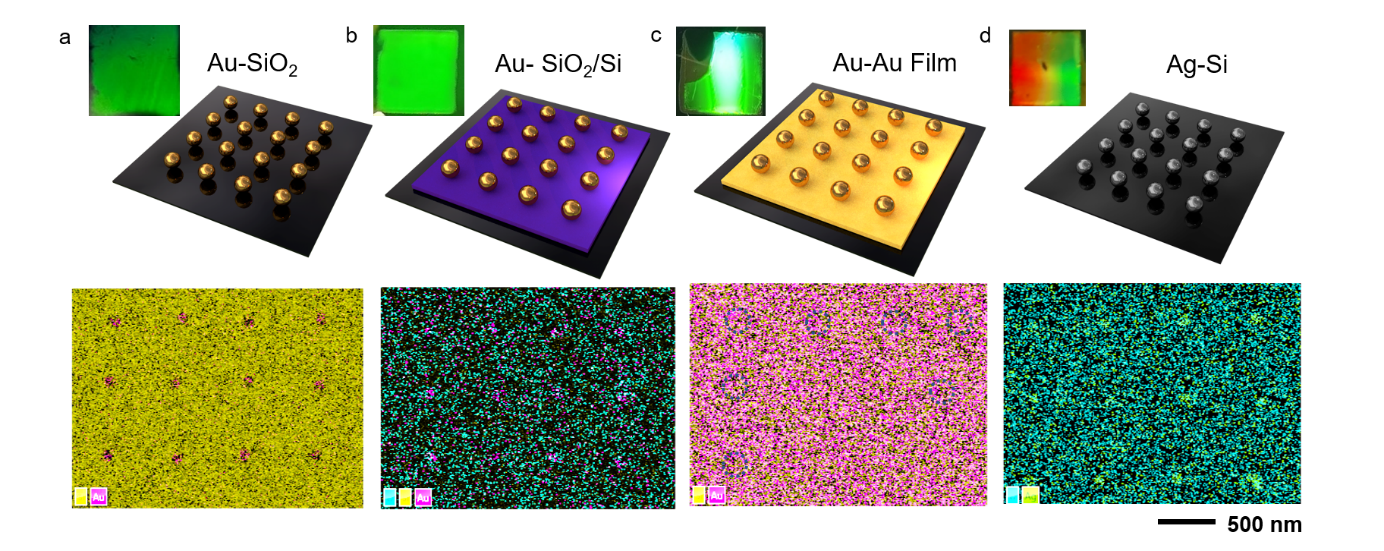


**Figure S25.** Characterization of NP arrays fabricated on various substrates and with different materials. (a-c) Photographs and corresponding Energy-Dispersive X-ray Spectroscopy (EDS) maps of AuNPs arrays on Si substrate (a) SiO_2_/Si substrate (b) and gold film substrate (c). (d) Photographs and corresponding EDS maps of Ag NP arrays on Si substrate.


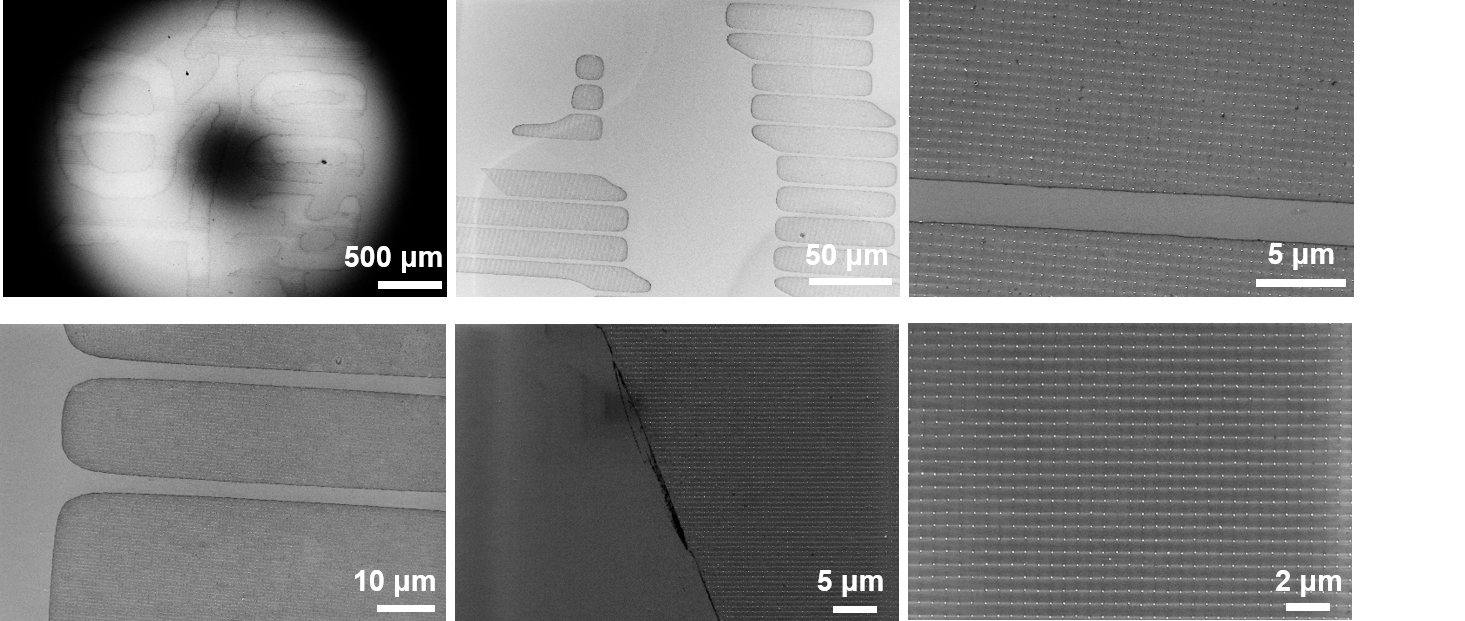


**Figure S26.** SEM of patterned AuNPs array in the emblem of Fudan University.


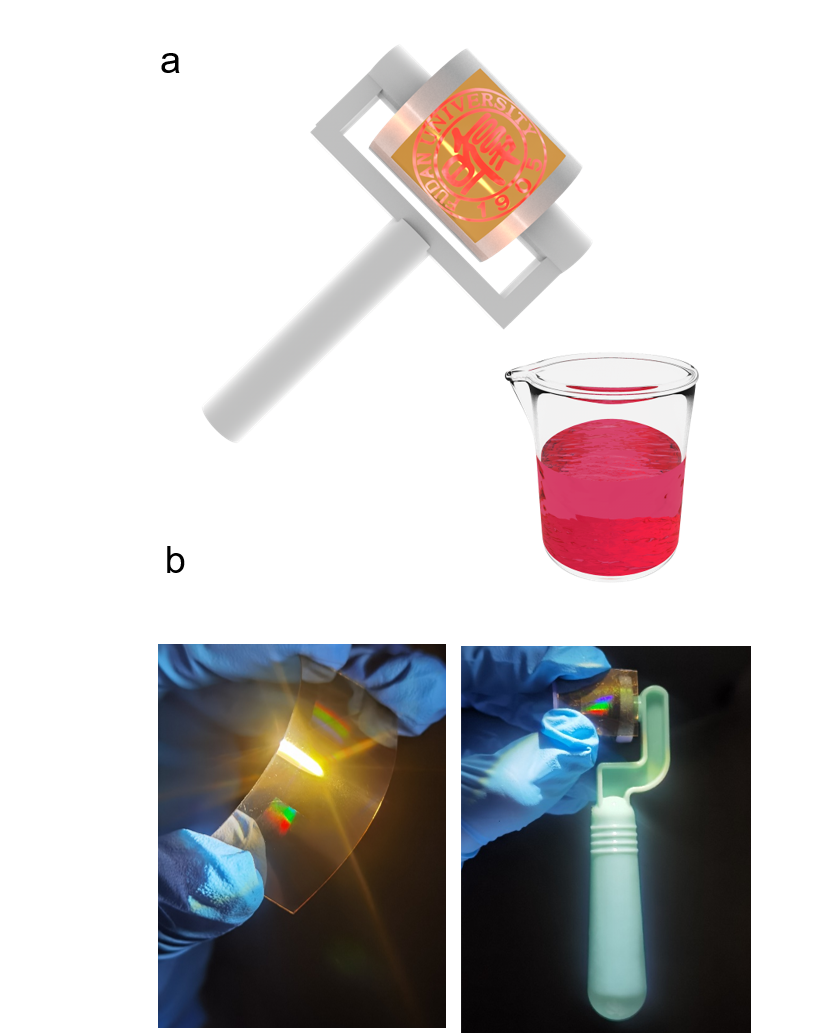


**Figure S27.** Continuous printing method using a stamp roller. (a)Schematic diagram of a continuous printing concept analogous to a stamp roller. In this analogy, a nanoparticle solution serves as the “ink,” and a patterned nanoparticle array prepared on a flexible substrate functions as the “stamp.” The properties of these nanoparticles, such as their size, shape, and composition, can be precisely controlled during synthesis, allowing for the creation of nanoparticle “ink” with tailored properties. The “stamp” in this process is a chemical patterned array which can peculiarly adsorb nanoparticle array prepared on a flexible substrate. The use of flexibility of the PET film enables the stamp to conform to the surface of the object being printed on, thereby ensuring a high-quality print. (b)Photograph of the AuNPs array on a Polyethylene Terephthalate (PET) film, and wrapped around the roller bearing.


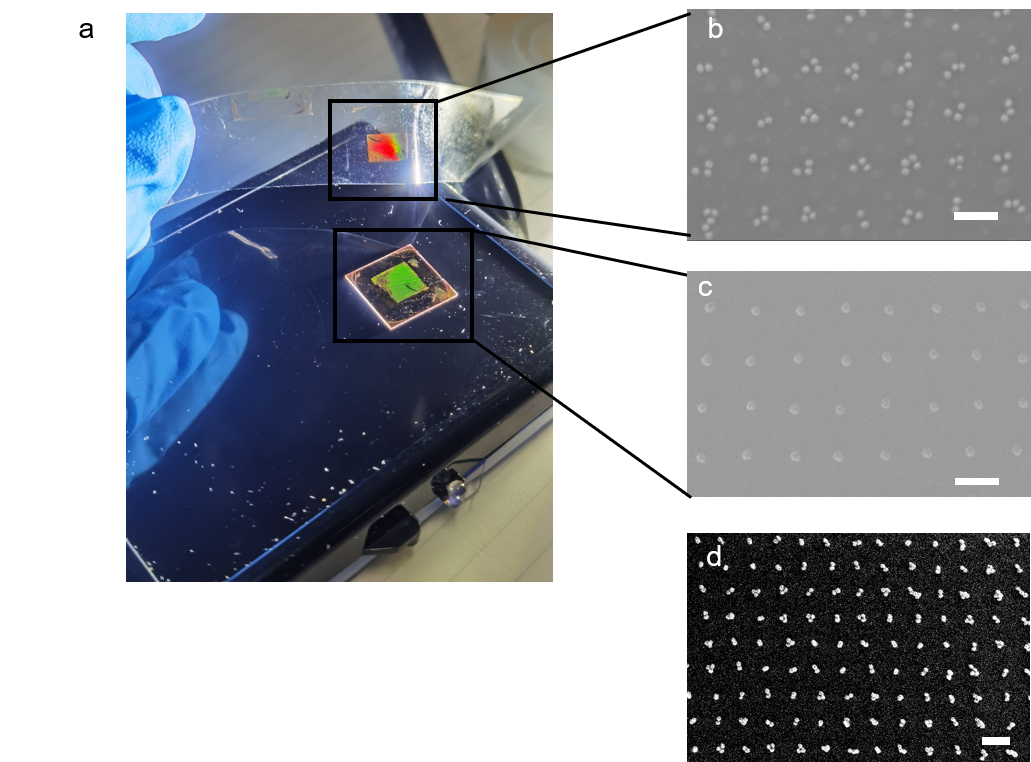


**Figure S28.** Characterization of second layer AuNPs arrays transferred onto tape from first layer AuNPs arrays. (a) Photographs of second layer AuNPs arrays transferred onto tape from the first layer of AuNPs arrays on quartz. (b) SEM image of second layer 50 nm AuNPs cluster array on the tape. (c) SEM image of first layer 100 nm AuNPs array on quartz. (d) SEM image of a second reabsorption of 100nm particles onto the first layer 100 nm AuNPs array. The first layer has the capability to absorb other particles and can be used repeatedly. Scale bar:500nm.

**Table S1.** The XPS element percentages of Si 2p, O 1s, N 1s and C1s of APTES chemical pattern array and Au 4f, O 1s, N 1s and C1s of P4VP chemical pattern array.

| **APTES-** **SiO_2_/Si** | **Si2p** | **O1s** | **N1s** | **C1s** |
| --- | --- | --- | --- | --- |
| **0 times** | 22.66 | 42.05 | 0.51 | 34.77 |
| **After tape silicon 30 times** | 26.08 | 54.09 | 0.51 | 19.33 |
| **After tape NP array 30 times** | 27.06 | 57.05 | 0.76 | 15.13 |

| **P4VP-Au** | **Au4f** | **O1s** | **N1s** | **C1s** |
| --- | --- | --- | --- | --- |
| **0times** | 39.02 | 17.48 | 5.39 | 38.1 |
| **After tape silicon 30 times** | 35.07 | 15.73 | 4.61 | 44.59 |
| **After tape NP array 30times** | 22.65 | 17.34 | 3.84 | 55.17 |

**Table S2.** Salt concentrations (*C*_NaCl_), colloid concentration (C*_NP_*), pH, and zeta potential of the NP dispersions utilized in fabrication.

| **Nanoparticles** | ***C*_NaCl_**  **[mM]** | ***C*_NP_**  **[mol/L]** | **pH** | **ζ potential**  **[mV]** |
| --- | --- | --- | --- | --- |
| **AuNPs_30 nm** | 0.033 | 3.1 × 10⁻^10^ | 5.85 | 32.9 ± 1.4 |
| **AuNPs_60 nm** | 0.066 | 3.2 × 10⁻^11^ | 5.75 | 33.8 ± 1.2 |
| **AuNPs_80 nm** | 0.05 | 1.8 × 10⁻^11^ | 5.85 | 42.4 ± 0.6 |
| **AuNPs_100 nm** | 0.1 | 1.4 × 10⁻^11^ | 4.96 | 29.2 ± 1.2 |
| **AuNPs_120 nm** | 0.1 | 2.8 × 10⁻^12^ | 4.51 | 28.5 ± 0.9 |
| **Ag NP_105 nm** | 0.1 | 2.4 × 10⁻^9^ | 3.52 | 23.5 ± 0.7 |
| **Ag NP_125 nm** | 0.1 | 1.3 × 10⁻^9^ | 3.56 | 29.5 ± 0.8 |
| **Ag NP_150 nm** | 0.1 | 9.7 × 10⁻^10^ | 3.59 | 27.1 ± 1.9 |

Supplementary Text

Calculation of the Position Error

import cv2

import numpy as np

import matplotlib.pyplot as plt

def in_witch_cell(point_x, point_y, cell_size_x, cell_size_y):

index_x = int(point_x / cell_size_x)

index_y = int(point_y / cell_size_y)

return index_x, index_y

# Read the original image and the template image

img = cv2.imread('image2_fixed.png', cv2.IMREAD_COLOR)

template = cv2.imread('template3.png', cv2.IMREAD_COLOR)

h, w, channels = template.shape

# Template matching

res = cv2.matchTemplate(img, template, cv2.TM_CCOEFF_NORMED)

threshold = 0.55

loc = np.where(res >= threshold)

points = np.array(list(zip(*loc[::-1])))

# Filter the recognized information

n = 10

filtered_points = []

for point in points:

if not any(np.linalg.norm(point - p) < n for p in filtered_points):

filtered_points.append(point)

filtered_points = np.array(filtered_points)

points_3d = np.zeros((14, 9, 2))

# Define the data scale, where 1 micron is equal to 133 pixels.

NORMAL_ONE = 133.0

step_size_x = 73

step_size_y = 81

start_x = 50

start_y = 26

# Locate the target in which cell and draw the object detection results

for pt in filtered_points:

cell_index_x, cell_index_y = in_witch_cell(pt[0],pt[1],step_size_x,step_size_y)

points_3d[cell_index_x][cell_index_y][0] = pt[0] + w/2

points_3d[cell_index_x][cell_index_y][1] = pt[1] + h/2

cv2.rectangle(img, tuple(pt), (pt[0] + w, pt[1] + h), (0, 255, 255), 1)

font = cv2.FONT_HERSHEY_SIMPLEX

font_scale = 0.30

color = (255, 0, 0)

thickness = 1

# Set the position of the grid

for x in range(start_x, img.shape[1], step_size_x):

cv2.line(img, (x, 0), (x, img.shape[0]), color, thickness)

for y in range(start_y, img.shape[0], step_size_y):

cv2.line(img, (0, y), (img.shape[1], y), color, thickness)

# color = (0,255,0)

# for x in range(0, img.shape[1], step_size_x):

# cv2.line(img, (x, 0), (x, img.shape[0]), color, thickness)

# for y in range(0, img.shape[0], step_size_y):

# cv2.line(img, (0, y), (img.shape[1], y), color, thickness)

# 0.55 is equivalent to 73 pixels, and 0.61 is equivalent to 81 pixels

size_x = 0.55

size_y = 0.61

errors = np.zeros((14, 9, 2))

errors_abs = np.zeros((14, 9, 2))

# Calculate and display the error for each cell

for i in range(start_x, img.shape[1], step_size_x):

for j in range(start_y, img.shape[0], step_size_y):

index_x = int((i - start_x) / step_size_x)

index_y = int((j - start_y) / step_size_y)

if index_y > 8:

break

gt_x = index_x * step_size_x + start_x

gt_y = index_y * step_size_y + start_y

sample_x = points_3d[index_x][index_y][0]

sample_y = points_3d[index_x][index_y][1]

dx = sample_x - gt_x

dy = sample_y - gt_y

dx = dx / NORMAL_ONE

dy = dy / NORMAL_ONE

errors[index_x][index_y][0] = dx

errors[index_x][index_y][1] = dy

errors_abs[index_x][index_y][0] = abs(dx)

errors_abs[index_x][index_y][1] = abs(dy)

#text = f"({( gt_x):.2f}, {( gt_y):.2f})"

text = f"({( dx):.2f}, {( dy):.2f})"

text_size = cv2.getTextSize(text, font, font_scale, thickness)[0]

text_x = i - text_size[0] // 2

text_y = j + text_size[1] // 2

color = (0,0,255)

cv2.putText(img, text, (text_x-9, text_y+7), font, font_scale, color, thickness)

x = errors_abs[:,:,0]

y = errors_abs[:,:,1]

# Draw an image of the absolute value of the error

plt.scatter(x, y)

plt.show()

cv2.imshow('image', img)

cv2.waitKey(0)

cv2.destroyAllWindows()
